# Interactive effects of warming and iron supplementation on O_2_ dynamics, trace metal content, and microbial diversity within different compartments of two Mediterranean corals

**DOI:** 10.1242/bio.062357

**Published:** 2026-01-20

**Authors:** Walter Dellisanti, Qingfeng Zhang, Elena Bollati, Davide Seveso, Christine Ferrier-Pagès, Caitlin Younis, Emma F. Camp, Michael Kühl

**Affiliations:** ^1^ Marine Biology Section, Department of Biology, University of Copenhagen, Strandpromenaden 5, 3000, Helsingør, Denmark; ^2^ Department of Earth and Environmental Science, University of Milano Bicocca, Piazza della Scienza 1, 20126, Milano, Italy; ^3^ MaRHE Center (Marine Research and High Education Center), Magoodhoo, Faafu, 12030, Maldives; ^4^ Coral Ecophysiology Laboratory, Center Scientifique de Monaco, Principality of Monaco, 98000, Monaco; ^5^ Climate Change Cluster, University of Technology Sydney, Broadway, NSW, 2007, Australia

**Keywords:** Coral holobiont, Mediterranean corals, Gastrovascular cavity, Microsensor, Microbiome restructuring, Trace metal metabolism

## Abstract

Mediterranean corals living in coastal habitats are subjected to natural fluctuations in temperature and nutrient availability, including substantial iron (Fe) inputs via terrestrial runoff (up to 14.5 nM). While Fe is essential for coral and symbiont metabolism, the assimilation rate, physiological thresholds, and spatial allocation of Fe within coral compartments, and its interactive effects with warming, remain poorly understood. Here, we provide the first characterization of oxygen (O_2_) dynamics, trace metal content, and microbial community composition in two Mediterranean corals, *Cladocora caespitosa* and *Eunicella singularis*, exposed to chronic warming (18-24°C) and Fe(III) supplementation (20 nM day^−1^). We show that although these corals are not Fe-limited, increased temperature enhanced the Fe uptake in the algal symbionts of *C. caespitosa*. In *C. caespitosa*, Fe supplementation reduced the O_2_ availability within the gastrovascular cavity (GVC) and altered the composition and diversity of GVC microbial communities. In *E. singularis*, interactive effects of Fe and warming reduced GVC O_2_ availability within the GVC, and warming increased metal content, while the microbiome resembled the surrounding seawater. These intraspecific differences in the sensitivity of the coral holobiont to warming and Fe supplementation could have important implications for the resilience of Mediterranean corals to ongoing climate stress, underscoring the importance of considering coral compartments in ecophysiological research.

## Teaser

**Summary:** Warming and iron supplementation reshape oxygen dynamics and microbiomes of the gastrovascular cavity of Mediterranean corals, revealing compartment-specific insights critical for understanding coral resilience under climate change.

## INTRODUCTION

Mediterranean corals thrive in dynamic environments characterized by high fluctuations in temperature, salinity, and nutrient availability, which shape their unique adaptability to changing environmental conditions (Bensoussan et al., 2010; Rivetti et al., 2014). This adaptability is increasingly critical as climate change accelerates the frequency and intensity of extreme events, such as marine heatwaves, which impose significant thermal stress on coral ecosystems worldwide (Frölicher et al., 2018; Hughes et al., 2018). In the Mediterranean Sea, prolonged and intense heatwaves have been documented to severely disrupt coral physiology and survival, posing a major challenge to their metabolic resilience and threatening the ecological balance of the habitats they form (Coma et al., 2009; Garrabou et al., 2009; Rodolfo-Metalpa et al., 2008b).

Given their exposure to natural thermal fluctuations, Mediterranean corals such as *Cladocora caespitosa* and *Eunicella singularis* serve as valuable models for investigating coral responses to ongoing and future climate change scenarios. *C. caespitosa* is one of the few endemic scleractinian coral species in the Mediterranean capable of forming biogenic structures, known as coral banks, which provide critical habitat for marine biodiversity (Kersting and Linares, 2019). This species exhibits high sensitivity to temperature anomalies, with mass mortality events linked to prolonged thermal stress and nutrient pollution, highlighting its vulnerability to extreme environmental conditions (Kersting et al., 2013; Quintano et al., 2025; Rubio-Portillo et al., 2018). On the other hand, the octocoral *E. singularis* contributes significantly to the structural complexity of Mediterranean benthic ecosystems. Despite its adaptability to varying thermal conditions, this species experiences tissue necrosis and partial colony mortality under severe and recurrent heat waves, emphasizing its susceptibility to increasing thermal stress (Ferrier-Pagès et al., 2009; Sarda et al., 2025).

Coral metabolism is profoundly influenced by the availability of micronutrients, which play critical roles in key cellular processes and macromolecule synthesis (Camp et al., 2022; Schoepf et al., 2015). Among these, iron (Fe) is particularly vital for the metabolic functions of both coral hosts and their photosynthetic symbiotic algae, as it is a cofactor in enzymes involved in many processes, particularly electron transport during photosynthesis and cellular respiration (Kroh and Pilon, 2020). However, Fe concentrations in seawater are often extremely low (<5 nM, GEOTRACES Intermediate Data Product Group, 2021), particularly in oligotrophic tropical marine environments where corals rely on efficient nutrient recycling within the holobiont to meet their metabolic demands (Sunda, 2012; Wang and Dei, 2003). In contrast, Mediterranean coastal waters experience intermittent but significant Fe supplementation (up to 14.5 nM) through terrestrial runoff, including riverine inputs, atmospheric deposition of Saharan dust, and anthropogenic sources such as industrial discharge (Bonnet and Guieu, 2004; Guerzoni et al., 1997; Guieu et al., 1997; Sarthou and Jeandel, 2001). These inputs can temporarily elevate Fe availability, influencing coral metabolic activity by enhancing the photosynthetic efficiency of symbiotic algae and supporting host cellular processes, such as carbon translocation, respiration, and growth (Moore et al., 2013). Enhanced Fe levels may also potentially improve the resilience of corals to thermal stress by supporting antioxidant defenses, although excessive Fe concentrations can lead to oxidative stress, potentially harming the coral holobiont (Dellisanti et al., 2024; Montalbetti et al., 2021; Leigh-Smith et al., 2018). Inorganic Fe is involved in redox cycling within coral tissue and associated microbial communities, fueling Fenton chemistry and reactive oxygen species (ROS) generation that can disrupt antioxidant defenses (Romero et al., 2022; Sandy and Butler, 2009). Changes in Fe availability can influence the functional complementation among other trace metals due to shared transport pathways, redox interactions, or binding sites (Rodriguez et al., 2016; Camp et al., 2022). Such cross-element interactions can lead to stoichiometric imbalances that affect enzymatic activity, oxidative stress responses, or microbial metabolism (Blanckaert et al., 2023).

In addition to its role in coral host and algal symbiont metabolism, increased Fe availability may influence the microbial communities residing within coral colonies (Rädecker et al., 2017). These microbes encompass protists, fungi, bacteria, and archaea, which all contribute to the remineralization of organic matter and the transformation of nutrients, such as nitrogen and phosphorus, into bioavailable forms that can be assimilated by the coral host or its symbiotic algae (Neave et al., 2016). The availability of Fe, a key factor in microbial metabolism, has the potential to significantly influence the composition and functionality of these microbial communities. For instance, increased Fe concentrations could enhance the activity of Fe-dependent enzymes involved in nitrogen cycling, potentially boosting processes like nitrification and nitrogen fixation in corals (Bourne et al., 2016; Moore and Doney, 2007). Such enhanced nutrient turnover may benefit the coral holobiont by providing additional nitrogen for metabolic needs and help keep overall nutrient accumulation in check to avoid the onset of bleaching (*sensu* Rädecker et al., 2021), especially under conditions of thermal or environmental stress. However, excessive Fe availability could also disrupt coral microbiomes, promoting opportunistic or pathogenic species that may negatively impact coral health (Bourne et al., 2016). The coral gastrovascular cavity (GVC) serves as a dynamic microhabitat where microbial interactions are tightly regulated to maintain homeostasis and metabolic efficiency, supporting processes like nitrogen fixation, nitrification, and denitrification (Agostini et al., 2012). In addition, changes in Fe availability can alter GVC O_2_ dynamics, further affecting coral health (Dellisanti et al., 2024).

Mediterranean corals may be particularly sensitive to shifts in microbial dynamics induced by seawater temperature changes, which can reduce the stress tolerance of the host and promote temperature-dependent bacterial diseases (Prioux et al., 2023). Importantly, elevated temperature can affect Fe metabolism by increasing metabolic and photosynthetic rates, increasing the cellular demand for Fe-dependent enzymes involved in electron transport, respiration, and antioxidant defense in corals (Shick et al., 2011; Tu et al., 2025). Temperature can also affect Fe bioavailability through changes in redox cycling and microbial Fe transformation processes, potentially altering the amount of bioavailable Fe accessible to the coral host and its symbionts (Sandy and Butler, 2009). Because temperate corals generally exhibit slower growth rates and increased sensitivity to environmental anomalies compared to tropical corals, they are important models for studying the complex interactions between micronutrients, microbial communities, and coral physiology in variable coastal ecosystems (Carbonne et al., 2024; Ferrier-Pagès et al., 2013; Kersting et al., 2013; Rubio-Portillo et al., 2018). Despite their exposure to fluctuating nutrients, how Fe depletion/supplementation and elevated temperatures interact to affect the physiology of Mediterranean corals remains unknown. This knowledge gap limits our understanding of how these corals, which differ in growth rates and thermal sensitivity, may respond to simultaneous increases in temperature and nutrient availability. In addition, the variability in terrestrial and atmospheric Fe inputs can modulate photosynthetic performance by optimizing electron transport efficiency and increasing O_2_ production (Guieu et al., 1997). Therefore, elevated Fe levels (>10 nM) in these coastal systems may provide Mediterranean corals with the necessary resources to meet the heightened metabolic demands imposed by increased temperatures, potentially improving their resilience to climate-induced stressors (Kersting et al., 2013, 2014; Rubio-Portillo et al., 2018).

In this study, we investigate the effects of chronic warming and inorganic Fe supplementation on the microenvironment of two Mediterranean coral species, *C. caespitosa* and *E. singularis*. We hypothesize that chronic low-level Fe supplementation (20 nM day^−1^) increases coral photosynthetic capacity and alters trace metal content in host and symbiont compartments; Fe supplementation increases microbial respiration and reduces O_2_ availability within the GVC; warming (+6°C above average) enhances the metabolic demand for Fe and alters Fe uptake in coral symbionts; the combined effects of warming and Fe supplementation disrupt physiological homeostasis and restructure microbial communities within coral compartments. Our findings show that although these corals exhibit resilience to warming and Fe supplementation at the holobiont level, Fe availability influences bacterial composition and activity within the GVC, providing new insights into the metabolic responses of temperate corals to trace metals and thermal stress.

## RESULTS

### Bulk photosynthesis-related responses to warming and Fe supplementation conditions

**Fig. 1.**
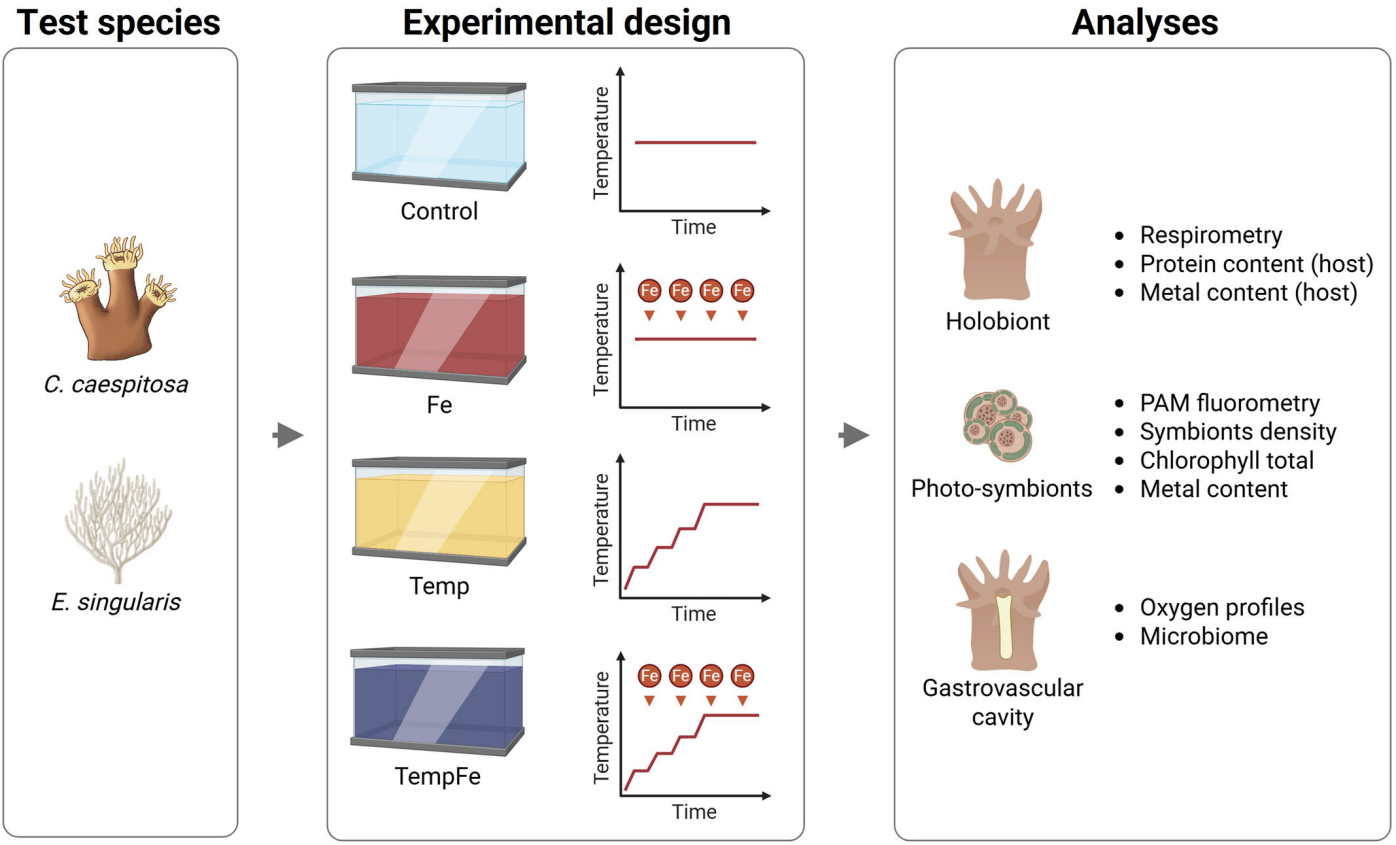
**Experimental design used in this study.** The coral species *C. caespitosa* and *E. singularis* were exposed to conditions of iron supplementation (Fe), increased temperatures (Temp), and a combined condition of iron supplementation and increased temperatures (TempFe). Physiological analyses were performed on the coral holobiont and their compartments, including photosymbionts, host tissue, and gastrovascular cavity. Created in BioRender by Dellisanti, W. (2025) https://BioRender.com/1ir4zpl. This figure was sublicensed under CC-BY 4.0 terms.

There were no notable changes in bulk O_2_ consumption and production, as both respiration and net photosynthesis in *C. caespitosa* and *E. singularis* remained stable over time, showing no significant response to increased temperature or Fe supplementation (Fig. 1, Fig. S2). Similarly, algal symbiont density, total chlorophyll concentration, and host protein content did not exhibit significant changes across treatments in either species (Fig. S3).

**Fig. 2.**
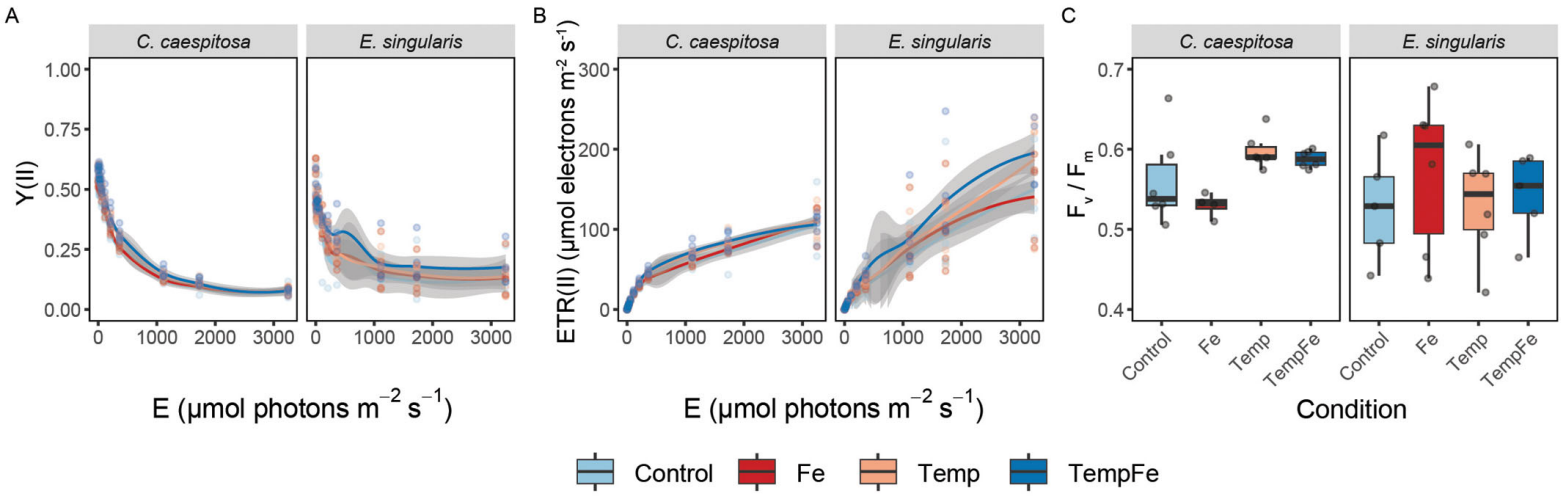
**Variable chlorophyll fluorescence measurements at the end of the experiment.** (A) Effective quantum yield of PSII, Y(II), and (B) derived relative electron transport rates, rETR, as a function of rapidly increasing photon irradiance (10 per irradiance step); (C) maximum photochemical quantum yield of PSII, F_v_/F_m_. Condition indicates the Control condition, the pulses of Fe(III) added to expose corals to 20 nM Fe day^−1^ (Fe), the increased temperature to 24°C (Temp), and the combined pulses of Fe and increasing temperatures (TempFe). The continuous line in panels A and B indicates the trend line using the *loess* method, with the shaded area as confidence intervals (95%).

Differences in variable chlorophyll fluorescence parameters were not evident across condition, species, and condition x species, particularly in the effective quantum yield of PSII, Y(II), and the derived relative electron transport rate, rETR, during rapid light curve measurements, or the maximum photochemical quantum yield of PSII (F_v_/F_m_) (Fig. 2, Table S1). Similarly, no significant changes in variable chlorophyll fluorescence parameters were observed under any condition in *E. singularis* (Fig. 2).

### Gastrovascular cavity

**Fig. 3.**
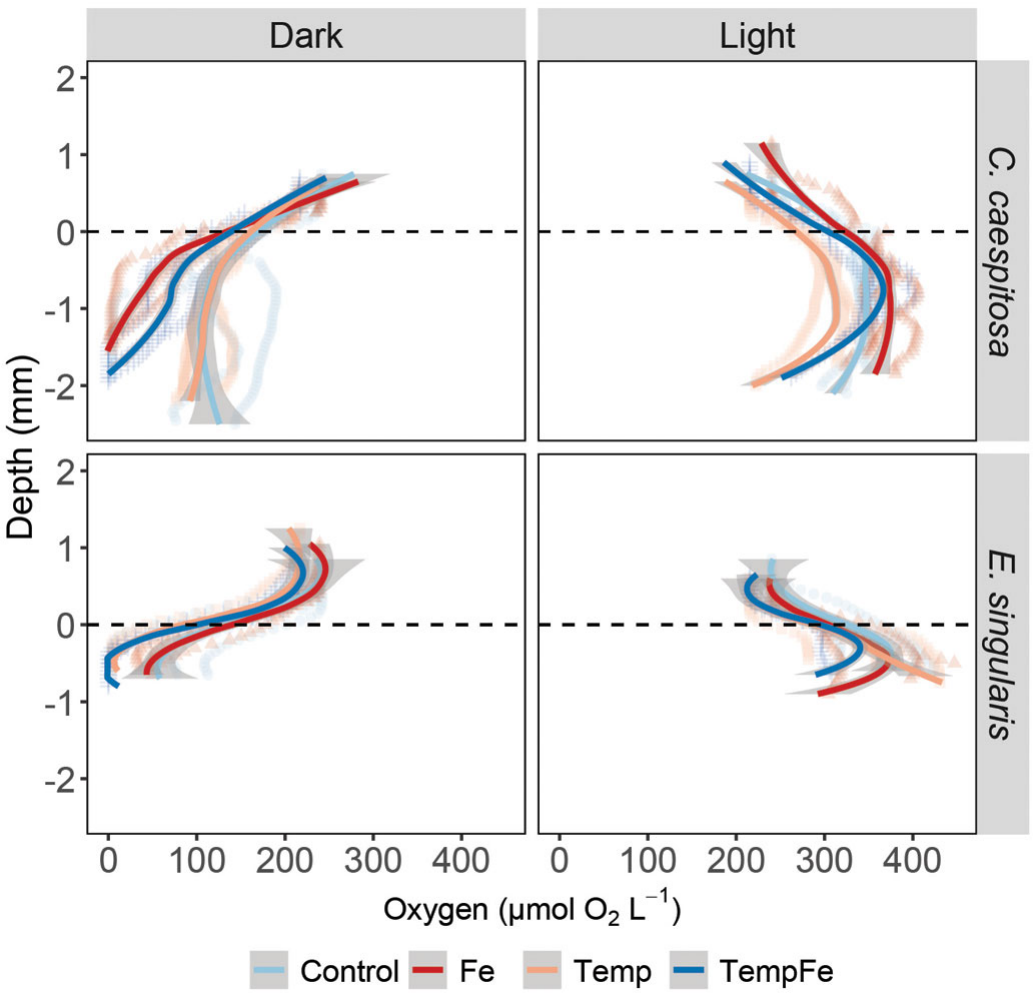
**O_2_ distribution and dynamics in the gastric cavity of coral specimens.** Depth profiles of O_2_ concentration in the gastric cavity of *C. caespitosa* and *E. singularis* were measured under dark and light (150 μmol photons m^−2^ s^−1^) conditions. Condition indicates the Control condition, the pulses of Fe(III) added to expose corals to 20 nM Fe day^−1^ (Fe), the increased temperature to 24°C (Temp), and the combined pulses of Fe and increasing temperatures (TempFe). The dotted line indicates the polyp mouth level, and the negative values indicate the depth within the GVC. The continuous line indicates the trend line using the *loess* method, with the shaded area as confidence intervals (95%).

The O_2_ availability within the gastric cavity of *C. caespitosa* and *E. singularis* exhibited significant differences across a depth range of 1 to 2 mm from the polyp's surface (Fig. 3, Fig. S3, Table S2). The availability of O_2_ in *C. caespitosa* under dark conditions was around 118±61.4 µmol O_2_ l^−1^, and this value did not change with increasing temperature (*P*=0.076). O_2_ availability decreased when corals were exposed to Fe supplementation, either alone or combined with increased temperature (TempFe), reaching 46.8±39.3 and 63.5±42.5 µmol O_2_ l^−1^, respectively (Kruskal–Wallis test, X^2^=71.4, d.f.=3, *P*<0.001). When *C. caespitosa* was exposed to light, we observed that exposure to Fe increased the levels of O_2_ in the gastric cavity to 365±22.6 µmol O_2_ l^−1^ (Kruskal–Wallis test, X^2^=130, *P*<0.001). For *E. singularis*, the availability of O_2_ in the gastric cavity in the dark was generally lower than for *C. caespitosa* (78.7±59.1 µmol O_2_ l^−1^), and it decreased further to reach hypoxic to anoxic conditions (28.8±33 µmol O_2_ l^−1^) when corals were exposed to increased temperatures or combined with Fe (26.5±32.5 µmol O_2_ l^−1^) (Kruskal–Wallis test, X^2^=20.8, *P*<0.001). When *E. singularis* corals were exposed to light, we observed that the exposure to combined Fe and temperature reduced O_2_ levels in the gastric cavity to 326±29.5 µmol O_2_ l^−1^ compared to Control 379.29±34.52 µmol O_2_ l^−1^ (Kruskal–Wallis test, X^2^=16.6, *P*<0.001).

### Metal content

**Fig. 4.**
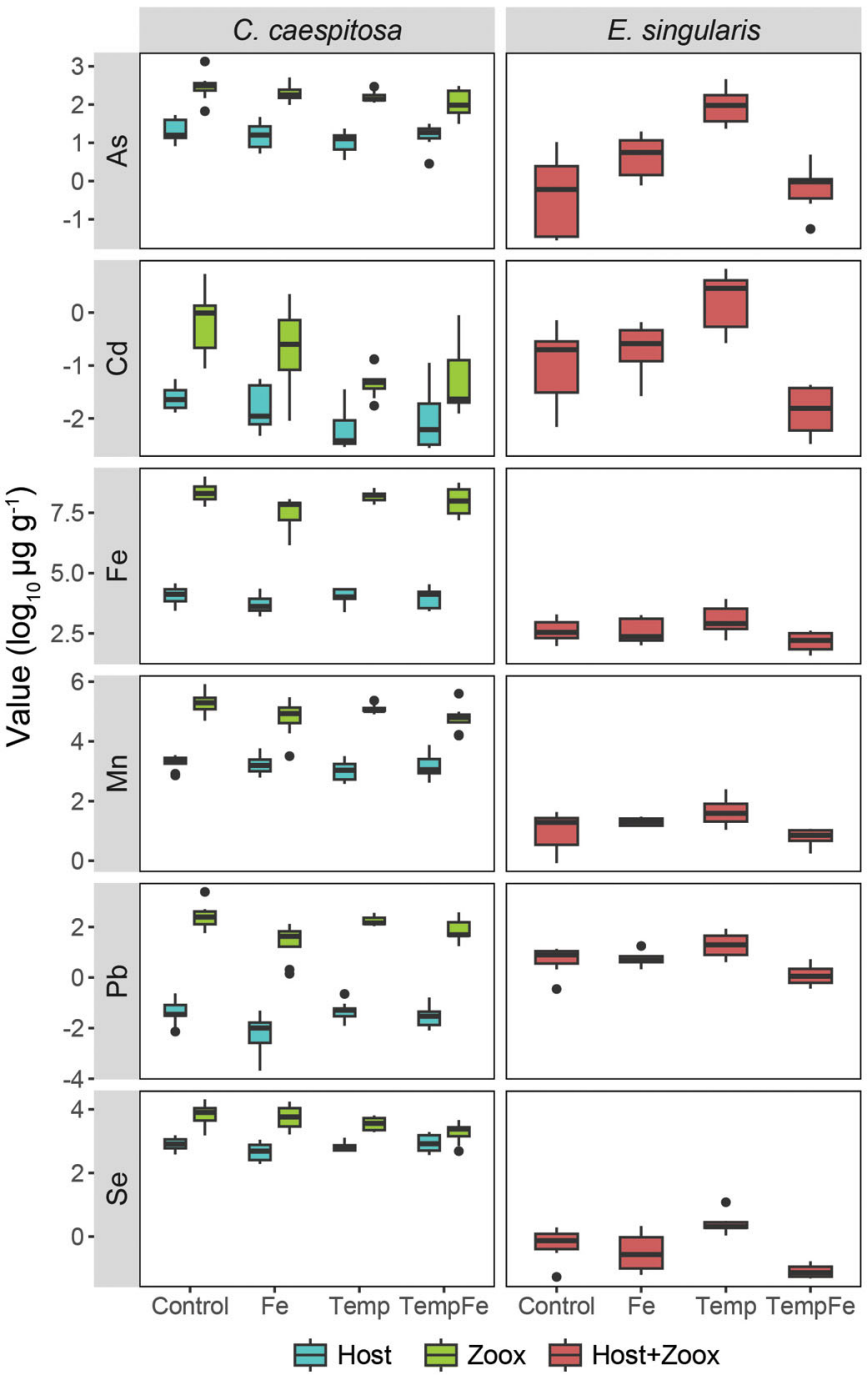
**Metallome profiles in *C. caespitosa* and *E. singularis* under experimental conditions.** Log_10_-transformed mean concentrations (μg g^−1^) of six trace elements in host tissue and symbionts. As, arsenic; Cd, cadmium; Fe, iron; Mn, manganese; Pb, lead; Se, selenium.

To assess how environmental conditions influence trace metal dynamics in coral holobionts, we quantified dissolved iron Fe(III) concentrations in seawater and analyzed metal accumulation in coral host and symbiont compartments. The levels of dissolved Fe measured in the tanks were 21.68±10.54 μg l^−1^, ranging between 31.31±12.42 μg l^−1^ in Control tanks and 10.51 μg l^−1^ in Temp tanks. The levels of Fe measured in seawater collected from the field at the sampling site were 40.86 μg l^−1^ (Table S3). Fe supplementation, temperature, and their combined treatment significantly affected the metallome of both *C. caespitosa* and *E. singularis* host tissue and symbionts (MANOVA, *F*=6.08, d.f.=3, *P*<0.001). However, the interaction between experimental conditions and origin (host versus symbionts) was not significant (Fig. 4, Fig. S5, Table S4). Among conditions, the temperature (Temp) treatment increased the metal content of *C. caespitosa* symbionts (Fe, Pb, Se) and *E. singularis* tissue (Fe, As, Cd, Mn, Pb), while reducing the Cd content in *C. caespitosa* symbionts compared to the host tissue. The combined effect of increased temperature and Fe supplementation (TempFe) also resulted in increased Fe content in *C. caespitosa* symbionts and Mn and Pb content in *E. singularis* tissue (Table S5).

### Microbiome composition and diversity

**Fig. 5.**
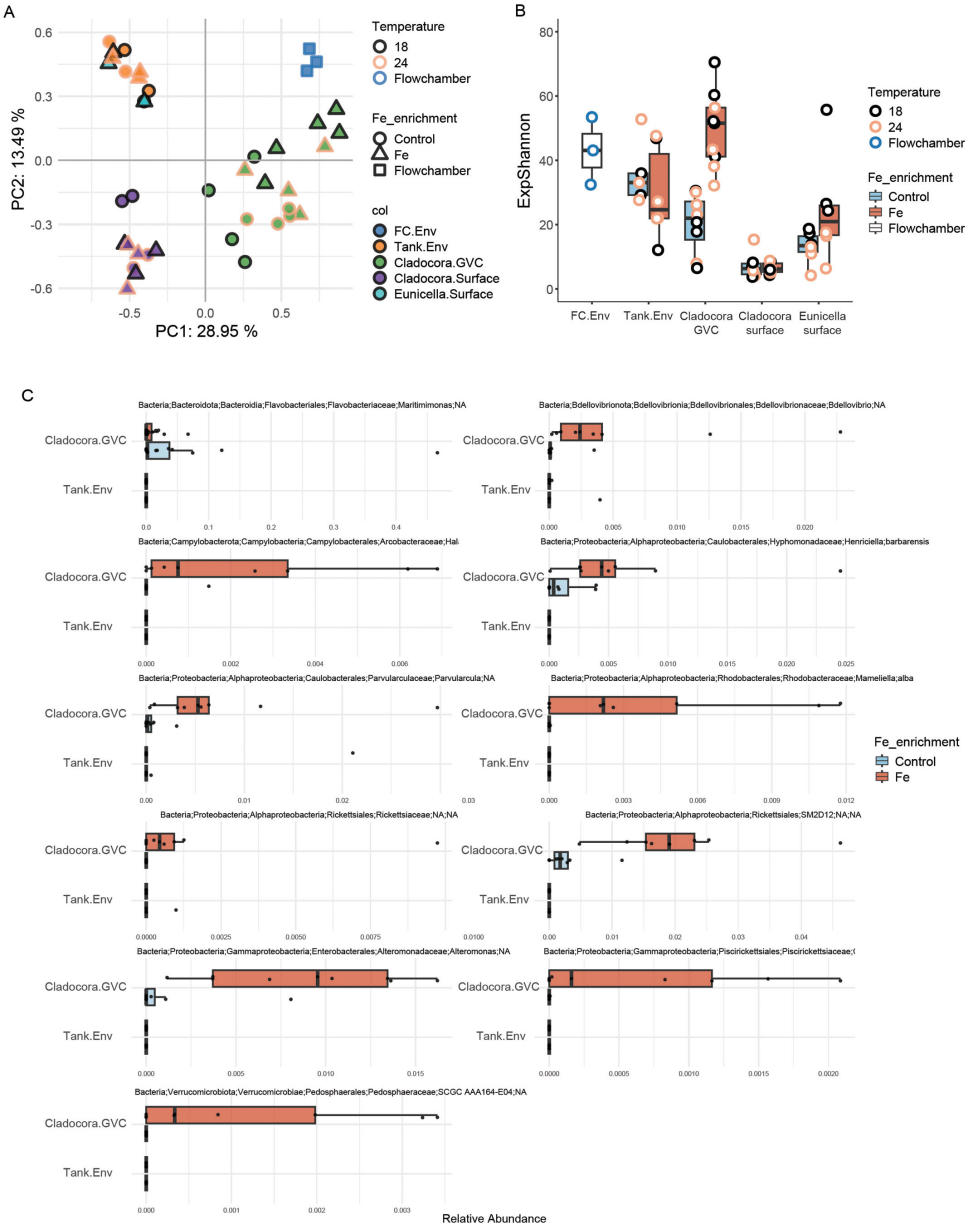
**Microbial diversity across coral compartments and environmental samples.** (A) Principal component analysis (PCA) based on Euclidean distance of clr-transformed data, showing bacterial community structure. (B) Alpha diversity is measured as the exponential of the Shannon index (e^H^). (C) Relative abundance of ASVs detected as differentially abundant between Fe and Control samples in the GVC of *C. caespitosa* (relative abundances in tank samples are shown for comparison).

Microbial community composition differed markedly across sample types (Fig. 5). PCA visualization of community composition showed a clear separation between the microbial communities associated with the different compartments of *C. caespitosa* (GVC and surface), which also appeared distinct from *E. singularis* and environmental samples (tanks and flow chamber). The *E. singularis* communities, on the other hand, clustered together with samples collected from the tanks (Fig. 5A). No clear clustering was observed between samples from the 18°C and the 24°C groups, however, some separation was evident between Fe-enriched and Control samples, at least in the *C. caespitosa* GVC (Fig. 5A). As 14 samples were flagged for having a high proportion of zeros, and thus removed from subsequent beta diversity statistics, we chose to increase statistical power by only interrogating the data for the effect of Fe. Additionally, we chose to focus on *C. caespitosa*, as the microsampling method does not allow for confident interpretation of host-associated samples that appear to closely resemble environmental samples, as observed here for *E. singularis* (see Discussion and Bollati et al., 2024).

For *C. caespitosa* samples, dispersion was heterogeneous between groups (*betadisper* followed by ANOVA, *F*_3,21_=14.1, *P*<0.001), with GVC samples showing higher dispersion compared to surface samples (*F*_1,23_=33.6, *P*<0.001). However, dispersion was even between the Fe and Control groups within surface (*F*_1,7_=0.6, *P*=0.46) and GVC (*F*_1,14_=1.0, *P*=0.34) samples. Stratified PERMANOVA indicated a significant effect of Fe supplementation on the *C. caespitosa* microbiome (R²=0.041, *F*=0.993, *P*=0.049); however, follow-up pairwise PERMANOVAs revealed that this effect was significant only within the GVC (R²=0.104, *F*=1.62, *adjusted P*=0.024), but not on the coral surface (R²=0.142, *F*=1.163, *adjusted P*=0.324) (Table S6).

Alpha diversity was significantly different between sample types (*C. caespitosa* GVC, *C. caespitosa* surface, *E. singularis* surface, and tanks; two-way ANOVA, *F*=28.71, d.f.=3, *P*<0.001) and between Fe-enriched and Control samples (*F*=8.08, *P*<0.01), and there was a significant interaction between these factors (*F*=4.73, d.f.=3, *P*<0.01). The effects of temperature were not tested, in line with the approach taken for beta diversity. Post hoc Tukey tests indicated that alpha diversity was higher in the GVC of *C. caespitosa* exposed to Fe supplementation, compared to surface-associated microbiomes and environmental samples (Table S7). In contrast, the microbial diversity in surface samples of both *C. caespitosa* and *E. singularis* was not significantly affected by Fe supplementation (Fig. 5B).

To investigate which microbial taxa may be driving the differences in microbiome structure and diversity detected in the GVC of *C. caespitosa* under Fe supplementation compared to controls, we performed differential abundance analysis on ASVs and aggregated taxa for these samples. Fe supplementation was associated with higher relative abundances of several ASVs within the GVC of *C. caespitosa* (Fig. 5C), including, among others, one *Bdellovibrio* (Bdellovibrionota; Bdellovibrionales), one *Parvularcula* (Alphaproteobacteria; Caulobacterales), *Mameliella alba* (Alphaproteobacteria; Rhodobacterales), *Halarcobacter bivalviorum* (Epsilonproteobacteria; Campylobacterales), *Henriciella barbarensis* (Alphaproteobacteria; Caulobacterales), and two species of Rickettsiales (Alphaproteobacteria). These taxa were absent or detected in low relative abundance in tank samples, regardless of Fe supplementation (Fig. 5C). Fe supplementation was also associated with reduced relative abundance of a *Maritimimonas* (Bacteroidota; Flavobacteriales) in the GVC of *C. caespitosa* (Fig. 5C). Analysis on taxonomically aggregated data revealed 18 significantly differential abundant taxa (Fig. S6); a relative increase in the orders Nannocystales and Sphingomonadales, the family Parvularculaceae, which was also abundant in the Fe-treated tank, and the genera *Marinobacter*, OM27 Clade, and *Parvularcula*, was detected in Fe-treated corals, while a decrease was detected for the genus *Maritimimonas* (Fig. S6). The common coral symbiont genus *Endozoicomonas* was present and highly abundant (>40%) in a single sample collected from the surface of *E. singularis*.

## DISCUSSION

Understanding the mechanisms that enable Mediterranean corals to persist across spatial and temporal scales is a critical objective in temperate reef ecology. This study explored how co-existing temperate coral species respond to ocean warming and chronic Fe supplementation, in terms of holobiont physiology, metallome, and microbial community composition. Our findings revealed species-specific responses in elemental composition, as well as changes in O_2_ dynamics and microbiome composition within the gastric cavity, but not at the holobiont level. Our results underscore the complexity of coral responses to environmental changes and highlight the importance of studying physiological and microbial processes at ecologically relevant spatial scales.

### No detectable effects of warming and Fe supplementation on the bulk physiology of corals

The temperature range used in this study (18-24°C) is below the bleaching level caused by marine heatwaves in the NW Mediterranean Sea (Tejedor et al., 2024; Vergotti et al., 2024). This level may stimulate the photosynthetic efficiency of the coral symbiotic algae (Symbiodiniaceae) (Hadjioannou et al., 2019), by enhancing PSII quantum yield and the activity of Fe-dependent electron transport enzymes, rather than increasing absolute photosynthetic rates alone (Raven et al., 1999; Reich et al., 2020), although this was not observed in our measurements. The F_v_/F_m_ values measured in *C. caespitosa* in this study (0.51-0.60) are comparable to those previously reported under Fe supplementation alone (0.54-0.63; Dellisanti et al., 2024), indicating that the corals maintained photochemical efficiency within the typical range for Mediterranean populations. This might be attributed to the species of Symbiodiniaceae hosted in *C. caespitosa* (*Symbiodinium microadriaticum*; Casado-Amezúa et al., 2014), known to be tolerant to temperature fluctuations (Iglesias-Prieto et al., 1992). However, the reduction in PSII quantum yield under Fe supplementation alone suggests that chronic Fe supplementation may disrupt photosynthetic processes, possibly due to an imbalance in electron transport or oxidative stress, although at lower levels than previously recorded for this symbiont species (Dellisanti et al., 2024, 2025). Similarly, the gorgonian *E. singularis* showed no significant changes in photosynthetic parameters under any tested conditions. The F_v_/F_m_ values measured here (0.42-0.68) fall within the range previously reported for this species under moderate thermal stress (0.40-0.65; Ezzat et al., 2013). Higher variability in variable fluorescence measurements in this species likely reflects the irregular morphology of gorgonian colonies, which complicates consistent single-point assessments using the standard glass-fiber optic probe of the PAM fluorometer. Moreover, gorgonian corals are considered more resistant to temperature fluctuations compared to scleractinian corals like *C. caespitosa* (Sarda et al., 2025; Viladrich et al., 2017). This resistance might be linked to their symbiotic association with *Philozoon*, a dinoflagellate genus characteristic of temperate regions (LaJeunesse et al., 2022; Sarda et al., 2025). The stability of photosynthetic performance in *E. singularis* under warming conditions may therefore reflect the physiological adaptations to temperature variability in temperate environments (Ferrier-Pagès et al., 2009). However, we acknowledge that the replication levels used in this study may limit the sensitivity for detecting changes in the bulk physiology of corals. Future studies should include expanded replication levels to help validate the effect size patterns identified here.

### GVC O_2_ dynamics

The measurement of O_2_ availability in the internal environment of *C. caespitosa* and *E. singularis* provided novel insights into compartment-specific photosynthesis and respiration dynamics under warming and Fe supplementation. The O_2_ level in the GVC of both corals in Control conditions ranged from ∼100 to 300 µmol O_2_ l^−1^ (in dark and light, respectively), consistent with previous findings in Mediterranean corals (Dellisanti et al., 2024). These values, however, are in contrast with the hypoxic (<50 µmol O_2_ l^−1^) to anoxic conditions reported in the GVC of tropical corals such as *Galaxea fascicularis*, *Dipsastraea favus*, *Coelastrea aspera*, and *Lobophyllia hemprichii* (Agostini et al., 2012; Bollati et al., 2024). This might suggest an enhanced capability of the Mediterranean corals to exchange O_2_ with the surrounding seawater. These differences might result from distinct polyp morphology, length, and contraction rates (Previati et al., 2010), as well as lower respiration rates indicative of reduced metabolic activity in temperate species compared to tropical corals (Reynaud and Ferrier-Pagès, 2019), which can affect the O_2_ availability within the gastric cavity. Chronic Fe supplementation, regardless of temperature, led to a decrease in O₂ levels within the GVC of *C. caespitosa*, while elevated temperatures alone were associated with significant reductions in O₂ levels in the GVC of *E. singularis*. Both species had hypoxic (<50 µmol O_2_ l^−1^) or anoxic GVCs under these conditions in the dark. These findings suggest that chronic exposure to Fe (for *C. caespitosa*) or increased temperature (for *E. singularis*) may enhance O_2_ consumption in either the coral tissue, the algal symbionts, the GVC microbiome, or a combination of these. Alternatively, the decreased O_2_ concentrations could be explained by a reduction in the exchange of water between the external and internal environments, leading to a build-up of hypoxic water in response to these stressors (Bollati et al., 2024; Dellisanti et al., 2024). Low O_2_ levels within GVC can induce oxidative stress by disrupting mitochondrial electron transport, increasing electron leakage and ROS formation, and activating hypoxia-responsive signaling pathways, as previously observed in corals (Alderdice et al., 2021, 2022; Dal Pizzol et al., 2022) and metazoans (Chandel and Schumacker, 2000). This can trigger various stress responses such as shifts in gene expression related to hypoxia tolerance (Bourne et al., 2016) or dysbiosis in the coral microbiome (La Rivière et al., 2016).

### Microbial communities

Bacteria residing within the GVC are emerging as a potentially critical community within the coral holobiont, with proposed roles in nutrient cycling, organic matter decomposition, and remineralization processes (Bollati et al., 2024; Bourne et al., 2016). Their metabolic activities can influence O_2_ availability and potentially mediate the bioavailability and metabolism of metals such as Fe (Thompson et al., 2014). Our *C. caespitosa* data support the notion that coral GVC microbial communities are distinct from those hosted in other anatomical compartments, i.e. the tissue surface mucus layer, not only in terms of species composition but also in terms of how they respond to environmental change. In this study, Fe supplementation significantly increased alpha diversity (indicating higher taxonomic and potential functional richness) and altered community composition in the GVC microbiome of *C. caespitosa* (Control: 20.4±9.2; Fe: 49.5±12), but not in the surface mucus layer. This could be a direct consequence of releasing Fe limitation, which may allow taxa with higher Fe requirements to colonize the GVC environment. While Fe requirements of individual taxa cannot be confidently inferred from 16S data, we note that the same *Parvularcula* ASV that increased in the GVC of Fe-treated *C. caespitosa* has been previously shown to grow on Fe-enriched media (D'Onofrio et al., 2010), suggesting that this taxon may have been directly stimulated by Fe availability in our experiment. Additionally, changes in the GVC microenvironment produced by host and/or symbiont metabolism may have mediated the effects of Fe on the GVC microbiome. Although no changes in overall holobiont photosynthesis and respiration were detected in bulk measurements, the drop in GVC O_2_ levels indicates a metabolic effect of Fe supplementation for at least one holobiont component in this compartment.

An increase in the availability of nutrients or labile carbon could create a more favorable environment for bacterial growth and colonization, and drive the relative increase in taxa like Rickettsiales, which are responsive to nutrient enrichment (Casas et al., 2004; Klinges et al., 2019). ASVs belonging to the genus *Bdellovibrio* and the closely related OM27 clade also increased in response to Fe treatment. These taxa are predators of gram-negative bacteria, so this relative increase could be driven by an increase in the availability or diversity of their prey in the GVC. Additionally, *Bdellovibrio* are particularly able to survive in high Fe environments and to digest Fe-rich prey thanks to their Fe(II) export system, analogous to vertebrate ferroportin and rare among bacteria (Bonaccorsi di Patti et al., 2015). An interesting result is the increase in some taxa associated with the Symbiodiniaceae phycosphere (*Mameliella alba* and the genus *Marinobacter*) in response to Fe supplementation. These bacteria are involved in complex metabolic interactions with coral symbionts and have been shown to enhance their growth in culture (Matthews et al., 2023; Ren et al., 2022). *Marinobacter* produces vibrioferrin, a Fe(III) binding siderophore, and could therefore contribute to increasing the bioavailability of Fe within the GVC for other bacteria and Symbiodiniaceae (Amin et al., 2009). In turn, exudates from dinoflagellates can stimulate the growth of *Marinobacter* (Amin et al., 2009). Further investigations into the complex interactions between host, algal symbionts, and prokaryotes within the coral GVC are needed to understand how changes in micronutrients such as Fe can impact the physiology of the holobiont. The GVC microbiome is smaller in biomass compared to communities found in other compartments (e.g. the skeleton), which means that it can be underrepresented in bulk sampling studies (Sweet et al., 2011), These subtle changes in response to Fe supplementation would likely have gone unnoticed using a bulk sampling approach. Our results, therefore, highlight the importance of studying interactions within the coral holobiont at ecologically relevant spatial scales.

A previous study on Mediterranean gorgonians has described a structured core microbiome that is predominantly composed of Gammaproteobacteria and Alphaproteobacteria, with a particular emphasis on the genus *Endozoicomonas*, as support of host homeostasis under stress (Rubio-Portillo et al., 2018; van de Water et al., 2017). Moreover, it has been proposed that temperate gorgonian corals harbor unique microbial communities adapted to oligotrophic conditions, which may buffer against rapid restructuring and associated physiological consequences (LaJeunesse et al., 2022). In our study, *Endozoicomonas* were only detected in a single *E. singularis* sample, and the overall community composition of the surface microbiome of *E. singularis* appeared to resemble that of the surrounding seawater. Our microsampling approach limits the interpretation of microbiome data when a community appears to be ‘environment-like’ (Bollati et al., 2024), as is the case here for *E. singularis*, because it cannot be established whether this is a true biological signal or the result of failed sampling and/or contamination. It is possible that the core microbes associated with *E. singularis*, previously detected with bulk-sampling methods (van de Water et al., 2017), reside within internal compartments, while surface samples are dominated by transient taxa present in the surrounding water. This would again point to the importance of sampling scales for understanding coral microbiomes.

### Effects on trace metal content

Trace metals, particularly Fe, are fundamental in the physiology of coral holobionts, influencing processes such as photosynthesis, enzymatic activity, and oxidative stress responses (Ferrier-Pagès et al., 2001; Harland and Brown, 1989; Rädecker et al., 2017; Raven et al., 1999; Reich et al., 2020). Other non-essential metals (such as Pb or Cd) are toxic to most life forms, and even essential trace metals such as Fe have adverse effects at elevated concentrations (Ali et al., 2011; Leigh-Smith et al., 2018). Thus, considering the metallome is important as the balance among essential, potentially toxic, and non-essential elements determines coral metabolic performance and stress resilience (Amorim et al., 2024; Camp et al., 2025; Reich et al., 2023).

Species-specific differences in metal content were expected between *C. caespitosa* and *E. singularis* due to their distinct biological and ecological characteristics (Peñuelas et al., 2019). In *C. caespitosa*, metallome analysis revealed that exposure to increasing temperatures, but not Fe supplementation alone, altered the metal content of photosynthetic symbionts, increasing Fe content from 2099.66 to 3467.54 μg g^−1^ and Pb from 4.81 to 9.37 μg g^−1^, while Cd concentrations decreased from 0.97 to 0.30 μg g^−1^. As a predominantly autotrophic coral, *C. caespitosa* highly depends on its photosymbionts, whose metabolic activities demand trace metals for key biochemical pathways. Changes in Fe content may represent an acclimation mechanism to support photosynthetic activity under stress (Dellisanti et al., 2024; Hadjioannou et al., 2019). However, the accumulation of lead (Pb) might indicate altered metal homeostasis under different stressors, which can lead to oxidative stress in both host and symbionts, and interfere with enzymatic activity (Montalbetti et al., 2021; Reichelt-Brushett and McOrist, 2003). Conversely, in *E. singularis*, increased temperatures resulted in higher levels of all trace metals measured in the coral holobiont. The flexible trophic strategy of this gorgonian coral, which relies less on photosynthesis and more on heterotrophy, likely drives its distinct metal uptake strategies. Sub-bleaching thermal conditions are known to stimulate the metabolic activity of *E. singularis* (Ezzat et al., 2013; Ferrier-Pagès et al., 2009), which could elevate the demand for metal-dependent enzymes and antioxidants that protect against stress. However, we could not distinguish whether these changes in metal content were related to photosymbionts or the host in *E. singularis* due to challenges in separating the holobiont components. Our findings align with previous studies on the role of local environmental conditions in shaping coral physiology and metallome composition (Camp et al., 2022). Moreover, Grima et al. (2022) highlighted the species-specific elementomes of scleractinian coral hosts and their associated Symbiodiniaceae, suggesting that symbiont elementomes are highly influenced by their metabolic needs and environmental conditions.

### Limited physiological benefits of Fe supplementation

While this study reveals compartment-specific responses to chronic Fe exposure, Fe supplementation under low-Fe baseline conditions did not confer measurable physiological benefits to either *C. caespitosa* or *E. singularis*. Although moderate levels of Fe can support the growth and cellular content development of cultured coral algal symbionts (Dellisanti et al., 2025), exposure to Fe induced a reduction in the O_2_ availability and restructuring of the microbial community within the GVC (this study). These effects suggest that moderate Fe inputs may increase oxygen demand through enhanced redox activity or microbial metabolism, potentially leading to localized oxidative or respiratory imbalances in specific coral compartments.

Differences between *C. caespitosa* populations from the north-west (NW) Mediterranean (this study) and the north-east (NE) Mediterranean Sea (Dellisanti et al., 2024) are likely to be due to variations in seawater temperature, nutrient availability, and metal concentrations. Corals from the NW Mediterranean Sea were collected from a nutrient-rich area with high metal accumulation in the sediments, which may have led to saturation of Fe requirements and reduced their response to further supplementation (Bonnet and Guieu, 2006; Guieu et al., 1997; Sarthou and Jeandel, 2001). In contrast, corals from the NE Mediterranean Sea were collected from a pristine site, and the limited Fe levels and dissolved metals might have made them more responsive to additional Fe supply (up to 50 nM), including Fe uptake, enhanced photosynthetic and respiratory activity, and higher minimum and maximum fluorescence yields of PSII (Dellisanti et al., 2024). These responses suggest that *C. caespitosa* corals in the NE Mediterranean are naturally Fe-limited compared to populations in the NW Mediterranean. Comparable patterns have been observed in tropical corals, where the exposure to desert dust enriched with trace metals, including Fe, enhanced the photophysiology of heat-stressed corals (Amorim et al., 2024). However, the effects of Fe alone were negligible, suggesting that the background nutrient and trace metal availability strongly modulates coral responses, and a combination of multiple metals may be more influential than Fe supplementation alone.

Fe plays a particularly important role in photosynthesis, serving as a cofactor in the electron transport chains of the symbiotic algae residing within coral tissues (Reich et al., 2020; Romero et al., 2022; Sandy and Butler, 2009). This pathway is essential for producing energy-rich compounds, such as ATP and NADPH, which fuel both the symbiont's cellular functions and the metabolic needs of the coral host (Raven et al., 1999). Fe-dependent enzymes, including cytochrome c and ferredoxin, mediate electron transfer processes critical for sustaining photosynthetic efficiency, particularly under environmental stress (Shi et al., 2010). However, interpreting these results remains constrained by key uncertainties: notably, the speciation and bioavailability of the added Fe(III), which may have been altered during dosing and exposure. The 20 nM daily supplementation is close to detection limits, but Fe(III) chemistry in seawater is highly dynamic, governed by rapid precipitation, organic complexation, and pH-dependent kinetics (Byrne and Kester, 1976; Johnson et al., 1997; Liu and Millero, 2002; Worsfold et al., 2014). Moreover, microbial cycling further modulates Fe bioavailability, as bacteria can reduce Fe(III) to the more bioavailable form Fe(II), or secrete siderophores that complex Fe(III) and facilitate uptake by both photosymbionts and other microbial taxa (Pi and Helmann, 2017; Sandy and Butler, 2009). These factors may have influenced Fe bioavailability in ways not captured by bulk seawater concentrations. Moreover, the short duration and single-mode delivery of Fe (as FeCl₃) may not reflect the complex, particulate, and colloidal forms encountered *in situ* through dust or runoff inputs. These limitations highlight the need for future experiments to integrate Fe speciation analysis, longer exposure durations, and more realistic delivery mechanisms to fully disentangle the functional role of trace metal pulses in coral holobionts.

### Conclusions

This study highlights the complex effects of warming and Fe supplementation on the physiology and metallome of Mediterranean corals, emphasizing compartment and species-specific differences in their responses. Fe supplementation alone may have caused stress by impairing O_2_ dynamics and restructuring the microbial communities within the GVC, and did not yield clear benefits for both *C. caespitosa* and *E. singularis*. These findings underscore the intricate balance between environmental conditions, coral physiology, and micronutrient availability. Future studies should address several critical gaps in understanding coral resilience to combined stressors. Long-term experiments are needed to assess the cumulative impacts of Fe supplementation and warming on coral growth, reproduction, and holobiont stability. Importantly, the gastrovascular cavity emerges as a functionally distinct and microbially dynamic niche that may mediate holobiont responses to environmental change (Hughes et al., 2022). Targeting this compartment in future research will be critical to understanding coral metabolic regulation and microbe–metal interactions under climate stress. Finally, integrating multi-stressor experiments that account for natural variations in nutrient availability and microbiome restructuring will be key to developing more realistic models of coral adaptation and survival in a changing ocean. These approaches will help inform conservation strategies, ensuring that interventions like nutrient supplementation or restoration efforts are tailored to the specific needs of different coral species and ecosystems.

## MATERIALS AND METHODS

### Sample collection and preparation

Three healthy colonies (10-30 cm^2^) of *C. caespitosa* with no visible sign of bleaching or disease were collected from a single site in Baia di Paraggi (Italy, 44°18′40.7″ N - 9°12′47.7″ E) using SCUBA in May 2023 (CITES IT/EX/2023/MCE/00335; AMP Portofino n.3/2023). Subsequently, the corals were carefully placed in a 50 l container with aerated seawater and transported to the Centre Scientifique de Monaco for laboratory experiments. Three colonies of *E. singularis* were originally sampled in May 2022 in a single site in Marseilles (France, 43°16′44.3″ N - 5°17′11.4″ E). Both species were kept in flow-through aquaria at the Centre Scientifique de Monaco that were continuously supplied with non-filtered Mediterranean seawater sampled from ∼50 m and heated to 18°C with salinity 37 ppt. *C. caespitosa* colonies were acclimated for 3 weeks, and *E. singularis* colonies had been maintained in the same flow-through system for 1 year. Corals were exposed to a 12:12 h light-dark cycle under a photon irradiance (400-700 nm) of 150±10 µmol photons m^−2^ s^−1^ provided by 400 W metal halide lamps (HQI-TS, Philips). Although natural Mediterranean photoperiods vary seasonally, a standardized 12:12 h regime is a widely adopted standard in coral ecophysiology to ensure consistent physiological baselines across treatments (Rodolfo-Metalpa et al., 2008a; Amorim et al., 2024). The light level was measured with a Universal Light Meter (ULM-500, Heinz Walz, Effeltrich, Germany). The coral colonies were fragmented into 96 nubbins (*n*=48 per species) and placed on PVC supports using epoxy resin putty. Nubbins of *C. caespitosa* consisted of single polyps (∼1 cm^2^), while those of *E. singularis* consisted of multiple polyps, with an approximate size of ∼3 cm^2^. The nubbins were then randomly transferred into eight experimental tanks (two tanks per experimental condition), with each tank containing a total of 12 nubbins (six nubbins per species), where they were allowed to acclimate for 2 weeks before the start of the experiments.

### Experimental settings

The experimental design consisted of four sets of two 20 l tanks for each experimental condition (Fig. 1): two tanks were maintained as Control conditions at 18°C without Fe(III) addition (hereafter named ‘Control’); two tanks were used for the Fe supplementation at 18°C and 20 nM Fe(III) day^−1^ (hereafter named ‘Fe’); two tanks were used for the warming treatment at 24°C without Fe(III) addition (hereafter named ‘Temp’); and two tanks were used for warming treatment at 24°C and Fe supplementation 20 nM Fe(III) day^−1^ (hereafter named ‘TempFe’). The tanks were filled with the same non-filtered seawater and maintained under controlled conditions in a water bath with a photoperiod of 12:12 and an incident photon irradiance (400-700 nm) of 150±10 µmol photons m^−2^ s^−1^. In the week prior to the start of the experiment, the temperature of the Temp and TempFe treatment tanks was raised by 1°C day^−1^ to reach the final temperature of 24°C. A stock solution of Fe(III) (100 µM FeCl_3_ • 6H_2_O, Sigma-Aldrich, St. Louis, MO, USA) was prepared for the Fe supplementation experiments. Two pulses of Fe(III) were added per day by diluting the stock solution to expose corals to 20 nM Fe day^−1^. A water change (40%) was carried out every day during the experimental period. The experimental conditions were maintained for 3 weeks. Coral in the Control condition were maintained under a low-Fe baseline relative to the field seawater. The level of Fe supplementation remained at or below concentrations measured at the collection site (∼40 µmol l^−1^, see Results section).

### Bulk photosynthesis parameters

The net production/consumption of dissolved O_2_ due to coral holobiont photosynthesis and respiration was measured using custom-made respirometry chambers (55 ml) composed of transparent polycarbonate (Dellisanti et al., 2024). The chambers were closed with a gas-tight transparent lid and contained a magnetic stirrer below a perforated plate at the bottom to ensure water mixing during incubation. An O_2_-sensitive optical sensor spot (OXSP5-ADH, Pyroscience GmbH, Aachen, Germany) was attached to each chamber's internal surface and was read out via a fiber optic cable (SPFIB-LNS, Pyroscience GmbH, Aachen, Germany) fixed via a holder on the outside of the transparent chamber wall and connected to a fiber-optic O_2_ meter (FSPRO-4, Pyroscience GmbH, Aachen, Germany). The meter was connected via a USB cable to a PC running data-logging software (Pyro Workbench; Pyroscience GmbH). Before experimental measurements, sensors were calibrated in µmol O_2_ l^−1^ using a 2-point calibration, measuring the sensor signals in anoxic (seawater with Na_2_SO_3_) and 100% air-saturated seawater at experimental temperature and salinity.

Dissolved O_2_ was recorded every second during the incubation of corals (*n*=4 per treatment per species, two per tank). Dark respiration (R) and net photosynthesis (P_n_) rates were calculated from the linear change in O_2_ concentration over time measured during 30-min dark (using blackout cloth, <5 μmol photons m^−2^ s^−1^) and 30-min light incubations using the *auto_rate* function from the R package *respR* (Harianto et al., 2019), respectively. Rates were calculated as (ΔO_2_/Δt)×V/A, where V is the volume (L) of seawater surrounding the coral samples in the chamber and A is the coral surface area (cm^2^), corrected with blank measurements (empty chambers). The coral surface and volume were determined by geometric approximations, measuring height and diameter of single polyps with an electronic caliper (precision=0.01 mm) (Naumann et al., 2009). Gross photosynthesis (P_g_) was calculated by adding the absolute value of R to P_n_, assuming that the dark respiration was identical to respiration in the light. Subsequently, P_g_:R ratios were calculated as a measure of the diurnal productivity and degree of autotrophy of the coral holobiont. We note that while P_g_ can be assigned to the photosynthesis of endosymbionts, R is affected by the respiratory activity of the coral host, its photosynthetic endosymbionts, as well as its microbiome.

### Variable chlorophyll fluorescence

The photosynthetic capacity of the endosymbionts in the coral samples was assessed with variable chlorophyll fluorimetry using a Pulse Amplitude Modulated (PAM) fluorometer (Dual-PAM, Heinz Walz, Effeltrich, Germany) equipped with a standard glass-fiber optic probe (Ralph et al., 1999). Single measurements were obtained from each polyp (*n*=5 per condition per species) at a 45-degree angle relative to the polyp surface after 15 min of dark acclimation. The maximum photochemical quantum yield of PSII (F_v_/F_m_) was calculated as (Schreiber, 2004): F_v_/F_m_=(F_m_−F_0_)/F_m_, where F_m_ is the maximum fluorescence yield measured during a strong saturation pulse (3000 µmol photons m^−2^ s^−1^, width 600 ms), F_0_ is the minimum fluorescence yield before the saturation pulse using weak measuring light pulses (<1 µmol photons m^−2^ s^−1^, width 3 µs, frequency 0.6 kHz), and F_v_ is the variable fluorescence calculated as F_v_=F_m_ – F_0_. The minimum fluorescence, F_0_, is measured when all PSII centers are open and can be used as a proxy for chlorophyll biomass (Serôdio et al., 1997), while the maximum fluorescence, F_m_, is measured when all PSII centers are closed in response to the saturation pulse (Baker et al., 2001). Rapid light curves (RLCs) were measured by illuminating dark-adapted corals at increasing irradiance from 0 to 3000 µmol photons m^−2^ s^−1^ (PAR; 400-700 nm) with 20 s incubation at each irradiance step (Ralph and Gademann, 2005; Trampe et al., 2011). The effective photochemical quantum yield of PSII was calculated as Y(II)=(F′_m_−F)/F_m_ (Genty et al., 1989) and provides a measure of the PSII photosynthetic capacity. The PSII relative electron transport rate (rETR) was calculated from the quantum yield of PSII, Y(II), and the actinic photon irradiance, E_d_, as rETR=Y(II)×E_d_ (Beer et al., 1998). All rETR, F_v_/F_m_, and Y(II) yields were calculated using the system software (WinControl, Heinz Walz GmbH, Effeltrich, Germany).

### Microsensor analysis

For microsensor measurements, coral nubbins (*n*=3 per condition per species) were placed inside a custom-designed flow chamber (0.8 l), with a consistent laminar water flow (0.5 cm s^−1^) of oxygenated seawater at experimental temperature (salinity of 35) as previously described (e.g. Haro et al., 2019). During light incubations, the corals were illuminated with a fiber optic white LED lamp (KL 2500 LED, Schott) with known photon irradiance (400-700 nm) levels of 150±10 µmol photons m^−2^ s^−1^, as determined by a Universal Light Meter (ULM-500, Heinz Walz, Effeltrich, Germany) equipped with a spherical micro quantum sensor (US-SQS/L, Heinz Walz, Effeltrich, Germany). The O_2_ microsensors were linearly calibrated from sensor signal readings in 100% air-saturated seawater and anoxic water (using a saturated Na_2_SO_3_ solution). The O_2_ microsensor was mounted on a motorized micromanipulator system (Unisense A/S, Denmark) and was connected to a multimeter (fx-6 UniAmp, Unisense A/S, Denmark). The micromanipulator and multimeter were connected to a PC running dedicated software for data acquisition and sensor positioning (SensorSuite Profiler v3.2, Unisense A/S, Denmark). The positioning of the microsensor tip relative to the coral surface was observed via a dissection microscope and a digital USB microscope (Dino-Lite 5MP Edge, AnMo Electronics, Taiwan). For measurements in the gastric cavity, one depth profile was measured per coral polyp. The microsensor tip was initially positioned at the center of the coral mouth, after which it was lowered vertically using the micromanipulator in 50-100 µm steps until a contraction of the coral polyp was observed. This position was identified as the base of the gastric cavity. After the microsensor signal was stable (minimum 10 min), depth profiles of O_2_ concentration were measured by moving stepwise from the cavity base toward the mouth and concluding (∼2 mm) in the seawater above the coral mouth.

### Symbiont density, chlorophyll, and protein concentrations

At the end of the experiments, nubbins were collected to determine algal symbiont density, chlorophyll *a* and *c2*, and protein concentration. The tissue of *C. caespitosa* nubbins (*n*=6 per condition) was separated from the skeleton using the water-pick technique using 9-26 ml of filtered seawater and a Potter tissue grinder for sample homogenization (Amorim et al., 2024). The tissue of *E. singularis* nubbins (*n*=6 per condition) was separated from the axis using a manual mortar and pestle and homogenized using an electric blender. Sub-samples of 50 µl of the slurry were used to quantify algal symbiont density using a LUNA FX7 automated Cell Counter (Logos Biosystems, South Korea). Another sub-sample of 5 ml was centrifuged at 5530×***g*** for 15 min at 4°C to separate host tissue and symbionts. The fraction containing algal symbionts (pellet) was used for chlorophyll analysis, while the fraction containing coral host tissue extracts (supernatant) was used for protein analysis. For chlorophyll analysis, the algal symbiont pellet was resuspended in 5 ml of pure acetone. The resulting solution was kept in the dark at 4°C for 24 h and then centrifuged for 15 min at 15°C and 5530 ***g***. Absorbance of the supernatant was read at 750, 663, and 630 nm on a spectrophotometer (UVmc2, Safas^®^). The equations from Jeffrey and Humphrey (1975) were employed to calculate chlorophyll *a* and *c2* concentration, which were then normalized to the skeleton surface area. For host protein quantification, proteins were extracted from the host fraction in a sodium hydroxide solution (1M for 30min at 90°C). Protein content was then quantified using a BC assay kit (Interchim) (Smith et al., 1985), and protein standards were prepared using bovine serum albumin (Interchim). The absorbance was measured at 562nm on a spectrophotometer (Xenius, Safas^®^).

### Metal analysis

Subsamples from the supernatant and algal symbionts extracted from coral nubbins (*n*=6 per species per condition) were freeze-dried and shipped to the University of Technology, Sydney, for metal analysis. Seawater samples (15 ml) were also collected at the beginning and the end of the experiment in metal-free tubes (Labcon ^®^) from the experimental tanks. The identification and quantification of elements present in the samples, including arsenic (^75^As), cadmium (^111^Cd), iron (^56^Fe), manganese (^55^Mn), lead (^208^Pb), and selenium (^78^Se), were performed by acid digestion followed by inductively coupled plasma mass spectrometry (ICP-MS) analysis on an Agilent ICP-MS 7700 following protocol of U.S. EPA (1994). A total of 250 µl Seastar ultrapure HNO_3_ and 250 µl of Seastar ultrapure H_2_O_2_ were added to each sample, and they were digested for 36 h. After digestion, 3.5 ml of high-purity water (Milli Q, Sartorius) was added to each sample to dilute the sample and acid before metal analysis. The metallome was determined using Agilent ICP-MS 7700, measured against Rh internal standard, and using H_2_ collision gas to avoid isotopic interference (U.S. EPA, 1994). On average, 30 mg of host tissue and 6 mg of algal symbiont fraction for *C. caespitosa*, and 13 mg of pooled material (host tissue with algal fraction) for *E. singularis* were used for ICP-MS analysis. Due to the complexity of separating zooxanthellae from the tissue, extracted samples of *E. singularis* were pooled, and the metal content was analyzed as a combined host–symbiont fraction. The final metal concentration was measured in 0.32 M HNO_3_ diluted samples using ICP-MS, and concentrations were expressed in µg L^−1^ or µg g^−1^ (U.S. EPA, 1994, 2014).

### Microbial community sampling

At the end of the experiment, microvolume samples for amplicon sequencing-based microbiome analysis were collected from corals (*n*=3 per species per condition). For both species, mucus samples were collected from the coral surface using a P10 pipette with a 10 μl pipette tip. For *C. caespitosa*, samples were also collected from the GVC using a sterile syringe equipped with a low dead-space microneedle (34G, 9 mm length, TSK, Canada), operated via the micromanipulator as described in (Bollati et al., 2024). 100 µl seawater samples were also collected from the tanks (*n*=3 per condition) and the flow chamber (*n*=3) using a P200 pipette. Samples were placed in sterile (UV-crosslinked for 1 h) 1.5 ml centrifuge tubes, snap frozen in liquid N_2_, and stored in a −80°C freezer. The samples were then transported on dry ice to the University of Copenhagen, where they were stored at −80°C until DNA extraction. DNA was extracted from all samples using a low-input (10 µl) protocol (Bramucci et al., 2021), as described in Bollati et al. (2024). The V3-V4 region of 16S rDNA was amplified using fusion primers with Illumina adapters (341 F: TCGTCGGCAGCGTCAGATGTGTATAAGAGACAG CCTAYGGGRBGCASCAG and 805 R: GTCTCGTGGGCTCGGAGATGTGTATAAGAGACAG GGACTACNNGGGTATCTAAT) in a 25 µl reaction mixture containing 5 µl of extracted DNA, 12.5 µl KAPA HiFi HotStart DNA ReadyMix (Roche, Basel, Switzerland), 0.5 µl of each 10 µM primer, and 6.5 µl PCR water. PCR amplification was performed under the following conditions: 98°C for 2 min; 35 cycles of 98°C:30 s, 55°C:30 s, and 72°C:30 s; followed by a final elongation at 72°C for 10 min. Amplicons were sent to the Australian Genome Research Facility (Melbourne, Australia), where they were purified, barcoded, and sequenced on Illumina NextSeq (300 bp paired-end, 633 total samples from multiple projects included in the run). Two extraction negative controls and two PCR negative controls were amplified and sequenced alongside the samples.

### Sequencing data processing

The demultiplexed reads were processed in R (v4.1.1) using the *dada2* pipeline (v1.22) (Callahan et al., 2016), after trimming adaptors and primers using *cutadapt* v4.4 (Martin, 2011). Forward and reverse reads were truncated at 250 bp, and the maximum number of expected errors was set to 2. Reads for the entire run were merged, denoised, and chimeras were removed, then the data were subset to remove samples from other projects. Taxonomy was assigned based on the Silva database v138.1 (Quast et al., 2013) with the default dada2 settings (Callahan et al., 2016). ASVs that made up more than 3% of total reads in either of the two extraction negative controls were removed from the dataset, together with any ASV detected in the two PCR negative controls. Finally, any ASVs identified as eukaryotic or originating from plastids were removed. The resulting dataset was used to plot rarefaction curves (Fig. S1) with the *vegan* v2.6-4 package (Oksanen et al., 2022). After inspecting the rarefaction curves, we deemed the sequencing depth to be satisfactory and chose not to rarefy the data.

### Statistical analysis

For data of bulk photosynthesis parameters, Rosner's generalized Extreme Studentized Deviate (ESD) test was used to detect the outliers in each measurement, combining all species and conditions, using the function *rosnerTest* of the R package *EnvStats* v3.0.0 (Millard, 2013). After the removal of the outliers (*n*=4), the data were log10 transformed, and the normal distribution of the data was verified with a Shapiro–Wilk test using the function *shapiro.test* from the R package *stats* v4.2.3 (R Core Team, 2023). Given that ‘respiration’, ‘net photosynthesis’, and ‘gross photosynthesis’ data did not follow a normal distribution, a generalized linear mixed model with a hierarchical structure (HLM) was used to evaluate condition effects by species. The model was fitted using the function *glmer* in the R package *lme4* v1.1-35.1 (Bates et al., 2015). The fixed factors for the model were ‘condition’ and ‘species’, while ‘tanks’ was a random factor. For P_g_:R calculation, a linear mixed model (*lmer*) with a Gaussian distribution was used to test the effects of the ‘condition’, ‘species’, and ‘tanks’ replicas. When data of bulk photosynthesis parameters and variable chlorophyll fluorescence did not meet the assumptions of normality, a Kruskal–Wallis test was used with the Wilcoxon rank test for pairwise comparison between experimental conditions.

A multivariate analysis of variance (MANOVA) was used to verify significant differences between ‘condition’ and ‘species’ groups in log10 variable chlorophyll fluorescence data, GVC O_2_ profiles, and metal content data, and visualized with a Principal Component Analysis (PCA). Differences in trace metal content were visualized using the function *radarchart* in the R package *fsmb* v.0.7.6 (Nakazawa, 2024).

Alpha diversity was calculated as Shannon's H index in *phyloseq* v1.52 (McMurdie & Holmes, 2013) and visualized as the exponential (e^H^). For statistical analysis, samples collected from the flow chamber were removed to obtain a balanced design. The data met assumptions of normality of residuals and homogeneity of variance (Shapiro–Wilk and Levene's tests, respectively), allowing ANOVA followed by Tukey's HSD post hoc comparisons. For beta diversity, a centered log-ratio (CLR) transformation was applied to mean-centered ASV counts after zero replacement using the count multiplicative approach (z.warning=0.9, frac=0.1) in *zCompositions* v1.5.0.4 (Palarea-Albaladejo and Martín-Fernández, 2015). Euclidean distance matrices were used for PCA and permutational multivariate analysis of variance (PERMANOVA), with stratification by sample type. Homogeneity of dispersion was tested using the *betadisper* function in the R package *vegan* v.2.7-1 (Oksanen et al., 2025), followed by ANOVA. Differential abundance analysis was performed pairwise to identify taxa of interest only in the GVC of Fe-treated versus Control *C. caespitosa*. The analysis was performed on both aggregated taxa and ASVs using ALDEx2 v1.26.0 (Fernandes et al., 2013). Taxa of interest were selected as those having unadjusted *P*<0.05 in ALDEx *t*-test, and effect size confidence intervals overlap <20%.

## Supplementary Material

10.1242/biolopen.062357_sup1 Supplementary information
