# Supplementary material for: Interactive effects of warming and iron supplementation on O2 dynamics, trace metal content, and microbial diversity within different compartments of two Mediterranean corals
Source: Biol Open. 2026 Jan 20;15(1):bio062357. doi: 10.1242/bio.062357 (PMC12869516; doi:10.1242/bio.062357)
Supplement: Supplementary information [file biolopen-15-062357-s1.pdf]

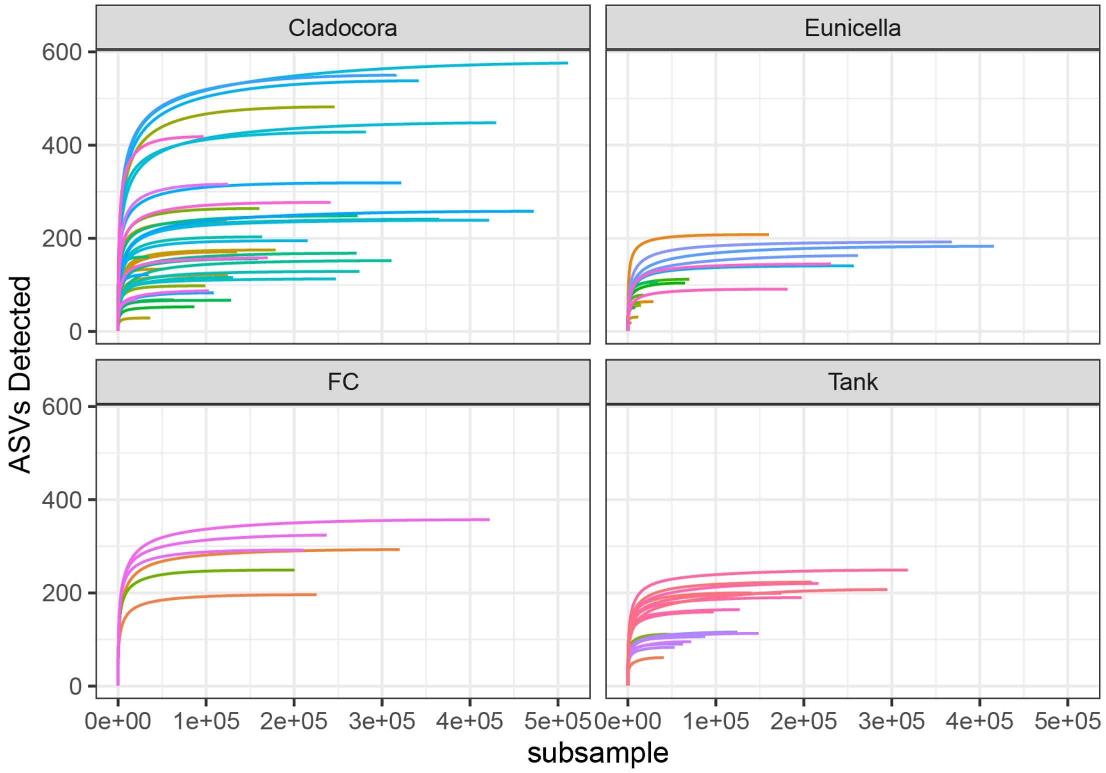

Fig. S1. Rarefaction curves showing the number of ASVs for each set of data from *Cladocora caespitosa*, *Eunicella singularis*, flow chamber (“FC”), and tank.

Biology Open • Supplementary information

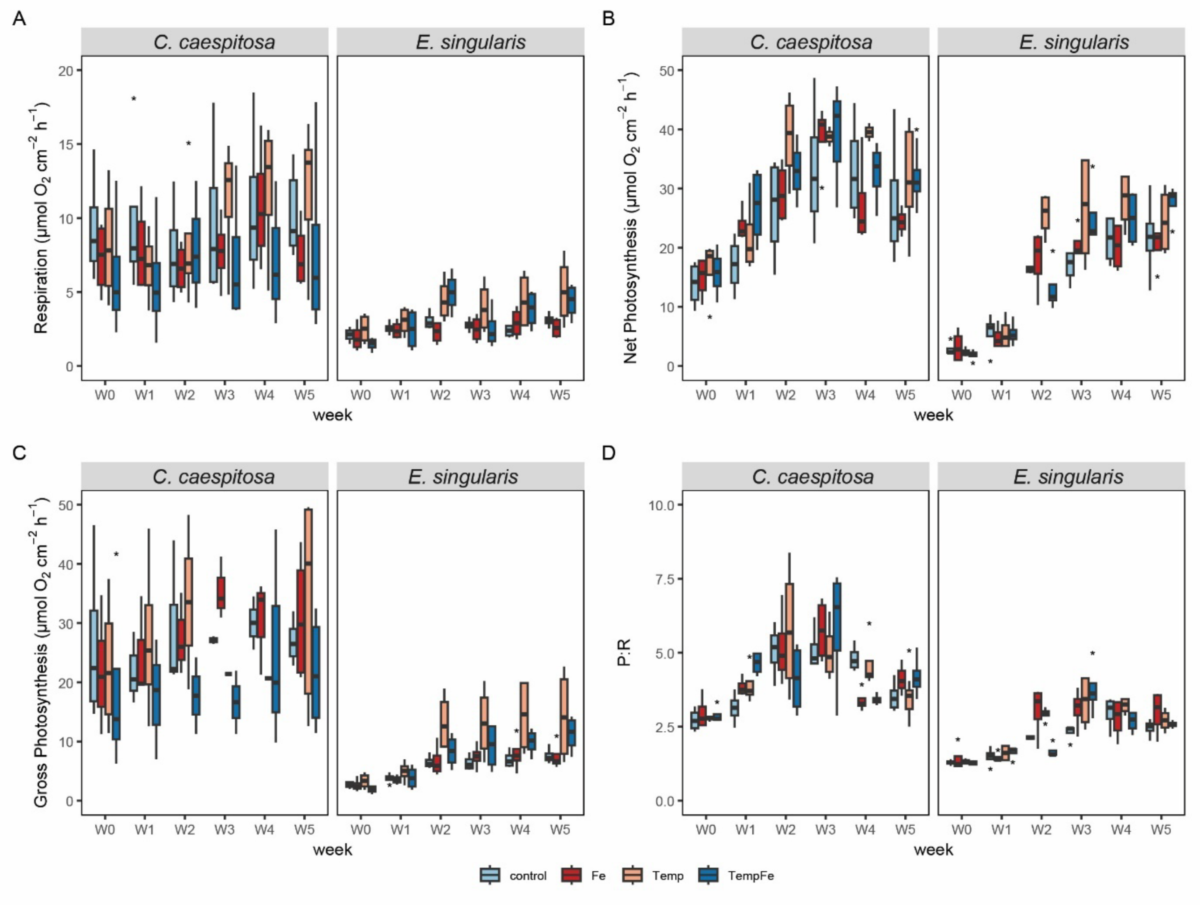

Fig. S2. Physiological parameters of corals under experimental conditions. (A) Respiration rate, (B) net photosynthesis rate, (C) gross photosynthesis rate, (D) ratio of gross photosynthesis to respiration (P_G:R) measured using respirometry in *C. caespitosa* and *E. singularis*. Condition indicates the Control, pulses of Fe(III) to expose corals to 20 nM Fe day^{-1} ("Fe"), increased temperature ("Temp") and combined exposure to high temperature and iron supplementation ("TempFe").

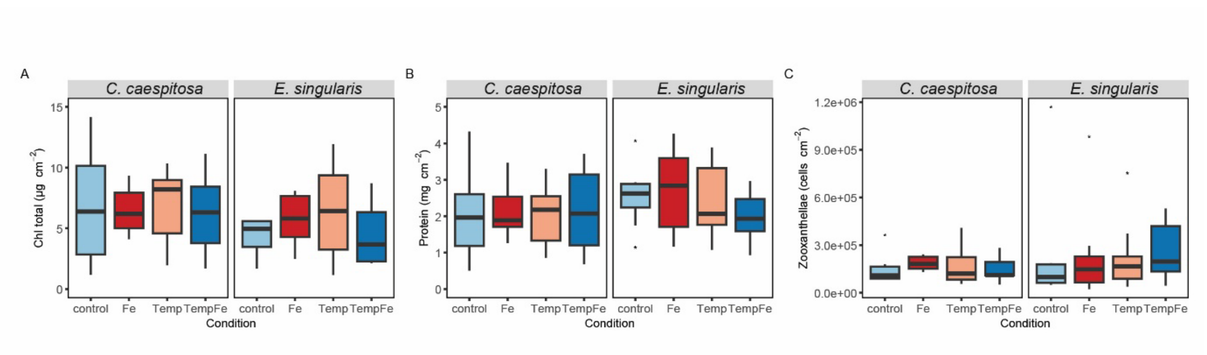

Fig. S3. Symbiont density, chlorophyll concentrations, and host protein content in *Cladocora caespitosa* and *Eunicella singularis* under different experimental treatments. Bar plots show (A) total chlorophyll (µg cm^{-2}), (B) host protein content µg cm $^{-2}$, and (C) symbiont density (cells cm^{-2}) for each species exposed to Control conditions (Control), iron supplementation (Fe, 20 nM Fe/day), elevated temperature (Temp, 24 °C), and combined stress (TempFe, 24 °C + 20 nM Fe day-1).

Biology Open • Supplementary information

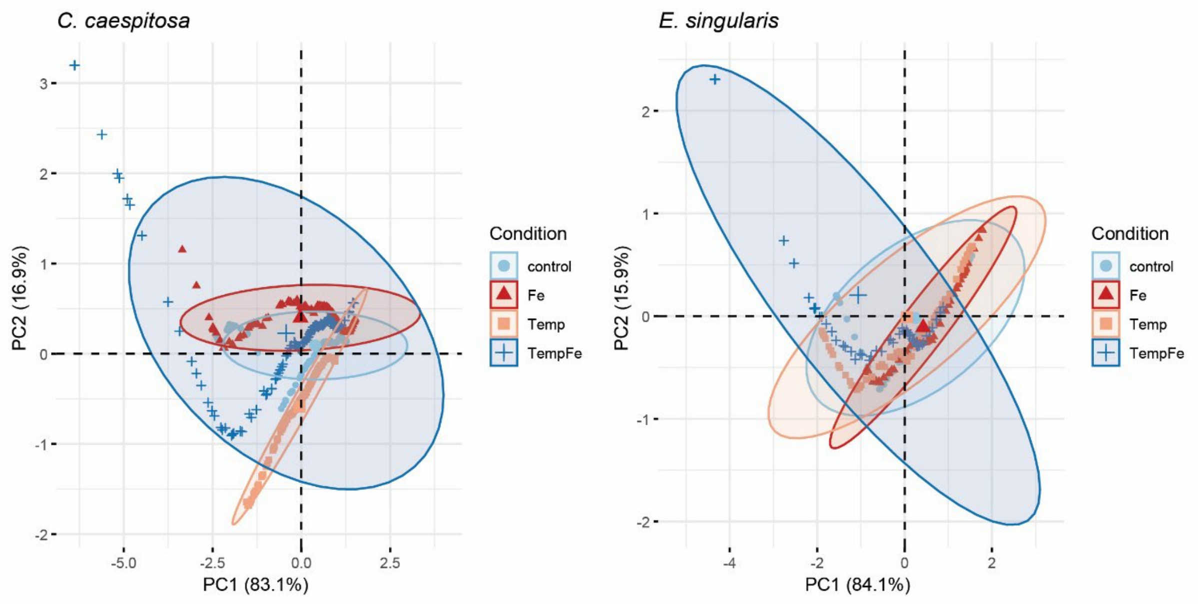

Fig. S4. Principal component analysis (PCA) of O_2 concentration profiles within the gastrovascular cavity of *Cladocora caespitosa* and *Eunicella singularis* under experimental conditions. PCA plots display variation in O_2 profiles across treatment groups: Control (light blue circles), iron supplementation (Fe, red triangles), elevated temperature (Temp, orange squares), and combined iron and temperature (TempFe, dark blue crosses). Ellipses represent 95% confidence intervals for each condition.

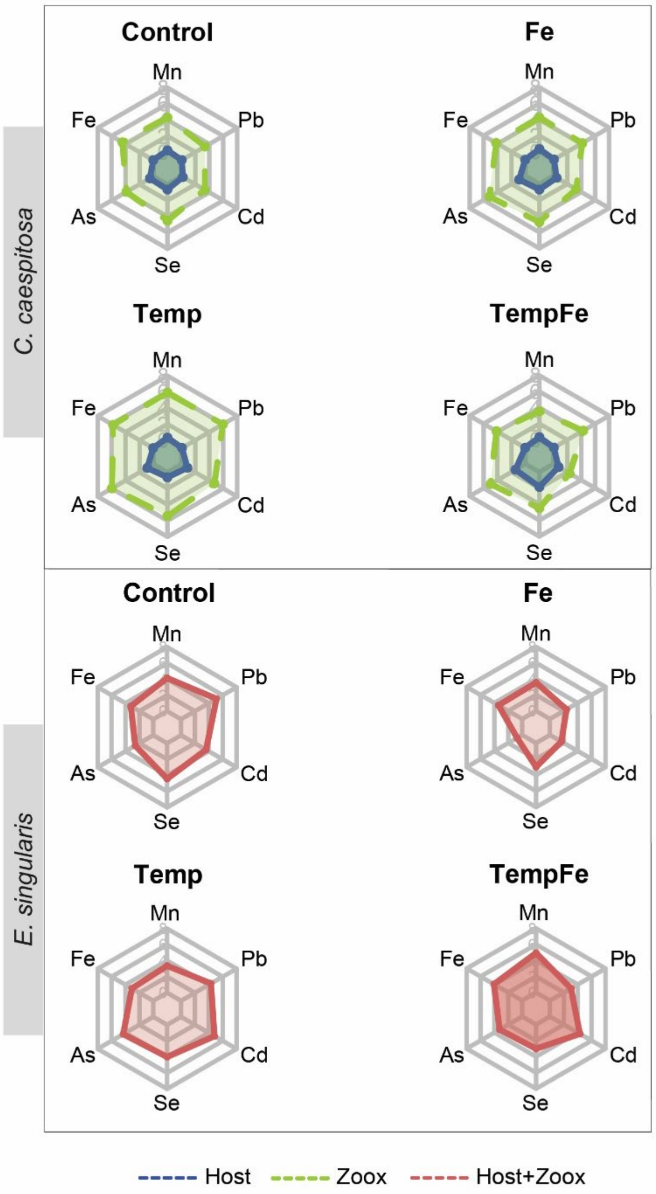

Fig. S5. Radar plots of log_{10}-transformed mean concentrations (µg g^{-1}) of six trace metals (Mn, Fe, As, Se, Cd, Pb) in coral host and symbiont compartments across treatments. Separate plots show metal content in the host tissue (blue) and algal symbionts (green). *Eunicella singularis* under the same four experimental treatments, where only the combined host-symbiont fraction was analyzed (red). The data represent treatment means. Maxima and minima were standardized per plot for visualization.

Biology Open • Supplementary information

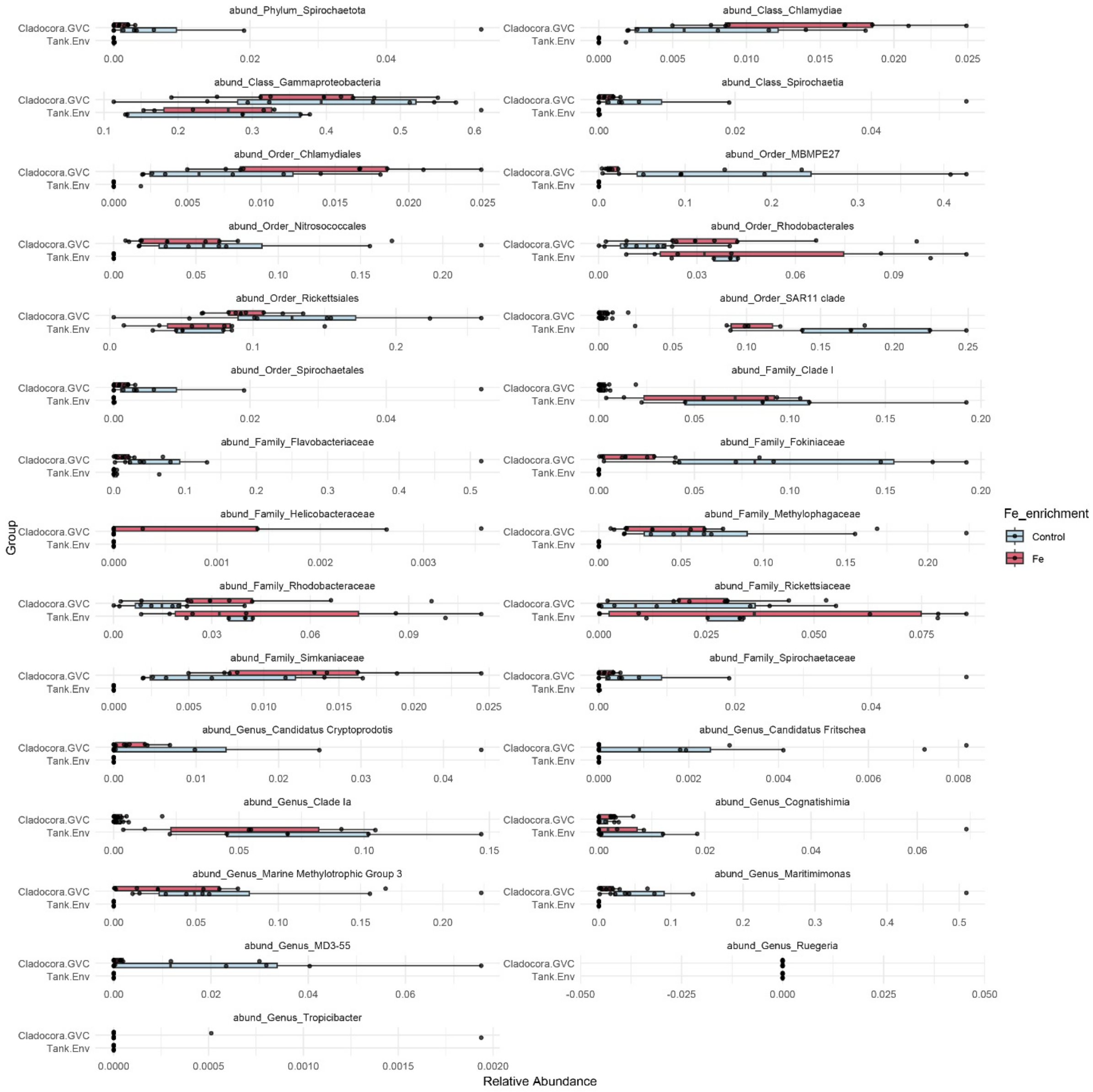

Fig. S6. Relative abundance of significantly enriched microbial taxa in *Cladocora caespitosa* gastric cavity and tank water samples under iron supplementation conditions (20 nM Fe/day). Taxa shown were identified as significantly different (ALDEx2, Benjamini–Hochberg *adjusted p* < 0.05, overlap < 0.2) across taxonomic levels (phylum to genus) and plotted as compositional relative abundance across sample groups (Cladocora.GVC, Tank.Water). Boxplots display the median and interquartile range with jittered individual sample points overlaid. Horizontal layout and facet wrapping by taxon facilitate comparison across groups. Iron supplementation did not universally increase microbial abundance but selectively modulated certain bacterial lineages. Colors represent treatment conditions: Control (blue) and Fe-enriched (red)

Biology Open • Supplementary information

Table S1. Multivariate analysis of variance (MANOVA) results for variable chlorophyll fluorescence parameters. The analysis tested the effects of experimental conditions (Control, Fe, Temp, TempFe), coral species (*Cladocora caespitosa*, *Eunicella singularis*), and their interaction. Df = degrees of freedom, Pillai = Pillai's trace, Approx. F = approximate F-statistic, and Pr(>F) = associated p-value.

**Multivariate analysis of variance (MANOVA)**				
	**Df**	**Pillai**	**Approx. F**	**Pr(>F)**
Condition	3	0.117	0.49	0.88
Species	1	0.147	1.96	0.14
Condition:Species	3	0.280	1.24	0.28
Residuals	36			

Table S2. Multivariate analysis of variance (MANOVA) results for O_2 concentration profiles within the gastrovascular cavity of *Cladocora caespitosa* and *Eunicella singularis*. The MANOVA tested the effects of experimental conditions (Control, Fe, Temp, TempFe), species, and their interaction on O_2 concentration profiles measured within the gastric cavity under light and dark conditions. Df = degrees of freedom, Pillai = Pillai's trace, Approx. F = approximate F-statistic, and Pr(>F) = associated p-value.

**Multivariate analysis of variance (MANOVA)**				
	**Df**	**Pillai**	**Approx. F**	**Pr(>F)**
Condition	3	0.235	38.994	< 0.001
Species	1	0.006	2.76	0.067
Condition:Species	3	0.137	21.945	< 0.001
Residuals	877			

Biology Open • Supplementary information

Table S3. Concentrations of trace metals (Mn, Fe, As, Se, Cd, Pb) in seawater, coral host tissue, and algal symbionts (zooxanthellae) under different experimental conditions. Data are reported as mean (± standard deviation) in µg L^{-1} for seawater samples and in µg g^{-1} dry weight for coral tissue and symbionts. Seawater samples include experimental tanks across all treatments (Control, Fe, Temp, TempFe) and a field reference. For *Cladocora caespitosa*, host and symbiont fractions were analyzed separately, while for *Eunicella singularis*, due to the difficulty of symbiont isolation, host and symbionts were analyzed as a combined fraction.

**Species**	**Compartment**	**Condition**	**Mn** µg L^{-1}	**Fe** µg L^{-1}	**As** µg L^{-1}	**Se** µg L^{-1}	**Cd** µg L^{-1}	**Pb** µg L^{-1}
Seawater	Tank	Control	0.76 (1.07)	31.31 (12.42)	0.15 (0.22)	16.50 (3.27)	0.00 (0.00)	0.00 (0.00)
Seawater	Tank	Fe	0.33 (0.02)	22.15 (2.85)	0.00 (0.00)	22.06 (2.97)	0.00 (0.00)	0.00 (0.00)
Seawater	Tank	Temp	0.00	10.51	0.00	24.39	0.00	0.00
Seawater	Tank	TempFe	0.00	12.62	0.00	16.28	0.00	0.00
Seawater	Field	Field	0.00	40.86	0.00	2.31	0.02	0.31
C. caespitosa	Host	Control	27.28 (5.94)	62.11 (21.95)	3.81 (1.10)	18.22 (3.69)	0.20 (0.04)	0.28 (0.14)
C. caespitosa	Host	Fe	26.23 (8.26)	44.68 (18.77)	3.51 (1.22)	14.95 (3.95)	0.18 (0.07)	0.14 (0.08)
C. caespitosa	Host	Temp	21.33 (6.57)	57.70 (16.12)	2.87 (0.78)	16.96 (2.37)	0.12 (0.05)	0.29 (0.11)
C. caespitosa	Host	TempFe	24.82 (10.57)	56.56 (22.16)	3.44 (0.88)	18.99 (5.10)	0.16 (0.12)	0.23 (0.10)
C. caespitosa	Zooxanthellae	Control	207.54 (81.43)	4534.55 (1933.74)	12.46 (4.82)	48.12 (15.80)	0.97 (0.56)	12.61 (7.54)
C. caespitosa	Zooxanthellae	Fe	134.59 (63.63)	2099.66 (1062.02)	10.39 (2.82)	44.93 (16.20)	0.64 (0.42)	4.81 (2.49)
C. caespitosa	Zooxanthellae	Temp	162.20 (24.98)	3664.42 (852.29)	9.24 (1.48)	35.05 (7.00)	0.28 (0.08)	9.62 (1.98)
C. caespitosa	Zooxanthellae	TempFe	127.32 (60.47)	3270.67 (1833.30)	8.07 (2.78)	27.52 (7.73)	0.33 (0.25)	7.11 (3.21)
E. singularis	Host+Zoox	Control	3.09 (1.61)	15.03 (6.92)	1.04 (0.97)	0.86 (0.36)	0.45 (0.27)	2.21 (0.90)
E. singularis	Host+Zoox	Fe	3.70	15.31	2.11	0.71	0.55	2.17

Biology Open • Supplementary information

			(0.50)	(8.05)	(1.06)	(0.42)	(0.23)	(0.66)
E. singularis	Host+Zoox	Temp	5.72	25.10	7.85	1.61	1.42	3.99
			(2.98)	(16.41)	(3.93)	(0.68)	(0.69)	(1.96)
E. singularis	Host+Zoox	TempFe	2.27	9.31	0.98	0.34	0.17	1.18
			(0.64)	(3.77)	(0.58)	(0.08)	(0.08)	(0.53)

Biology Open • Supplementary information

Table S4. Multivariate analysis of variance (MANOVA) results testing the effects of condition, coral species, and tissue origin on trace metal content. The MANOVA was performed on log-transformed concentrations of six trace metals (Mn, Fe, As, Se, Cd, Pb) in host tissue and algal symbionts of *Cladocora caespitosa* and *Eunicella singularis* across experimental conditions (Control, Fe, Temp, TempFe). Df = degrees of freedom, Pillai = Pillai's trace, Approx. F = approximate F- statistic, Num Df = multivariate numerator degrees of freedom, Den Df = multivariate denominator degrees of freedom, and Pr(>F) = *associated p*-value.

**Multivariate analysis of variance (MANOVA)**						
	**Df**	**Pillai**	**Approx. F**	**Num Df**	**Den Df**	**Pr(>F)**
Condition	3	1,327	6.08	18	138	< 0.001
Species	1	0.988	646.58	6	44	< 0.001
Origin	1	0.968	220.89	6	44	< 0.001
Condition:Species	3	0.986	3.75	18	138	< 0.001
Condition:Origin	3	0.329	0.95	18	138	0.525
Residuals	49					

Biology Open • Supplementary information

Table S5. Effects of iron supplementation (Fe), increased temperature (Temp), and their combination (TempFe) on metal content in the symbionts and host of *Cladocora caespitosa* and *Eunicella singularis*. Coloured cells indicate trends in metal concentrations across treatments and compartments (**Fig. 4**). Notes: *n.s.* (=): Not significant, no change observed; ↑: indicates an increase in metal content; ↓: indicates a decrease in metal content.

Cladocora caespitosa						
			**Symbionts**			
**Metal**	***Fe* 18 °C**		***Temp* 24 °C**		***TempFe* 24 °C**	
**As**	*n.s.*	=	*n.s.*	=	*n.s.*	=
**Cd**	*n.s.*	=	$p < 0.05$	↓	$p < 0.01$	↓
**Fe**	*n.s.*	=	$p < 0.01$	↑	$p < 0.01$	↑
**Mn**	*n.s.*	=	*n.s.*	=	*n.s.*	=
**Pb**	*n.s.*	=	$p < 0.01$	↑	$p < 0.01$	↑
**Se**	$p < 0.01$	↑	*n.s.*	=	*n.s.*	=
			**Host**			
**Metal**	***Fe* 18 °C**		***Temp* 24 °C**		***TempFe* 24 °C**	
**As**	*n.s.*	=	*n.s.*	=	*n.s.*	=
**Cd**	*n.s.*	=	*n.s.*	=	*n.s.*	=
**Fe**	*n.s.*	=	*n.s.*	=	*n.s.*	=
**Mn**	*n.s.*	=	*n.s.*	=	*n.s.*	=
**Pb**	$p < 0.05$	↓	*n.s.*	=	*n.s.*	=
**Se**	*n.s.*	=	*n.s.*	=	*n.s.*	=
Eunicella singularis						
			**Symbionts + Host**			
**Metal**	***Fe* 18 °C**		***Temp* 24 °C**		***TempFe* 24 °C**	
**As**	*n.s.*	=	$p < 0.01$	↑	*n.s.*	=
**Cd**	*n.s.*	=	$p < 0.01$	↑	*n.s.*	=
**Fe**	*n.s.*	=	$p < 0.05$	↑	*n.s.*	=
**Mn**	*n.s.*	=	$p < 0.05$	↑	*n.s.*	=
**Pb**	*n.s.*	=	$p < 0.01$	↑	*n.s.*	=
**Se**	*n.s.*	=	$p < 0.01$	↑	*n.s.*	=

Table S6. Permutational multivariate analysis of variance (PERMANOVA) results testing the effect of iron supplemenation on microbial community composition. The analysis was performed on CLR-transformed ASV data using Euclidean distance matrices. A stratified PERMANOVA (adonis2, 999 permutations) was conducted with sample type (Cladocora GVC vs. surface) as the strata. A follow-up PERMANOVA analysis was performed within each compartment.

Permutational Multivariate Analysis of Variance (PERMANOVA)

adonis2(formula = dist_matrix ~ Fe_supplemenation, data = samdata, strata = samdata$SampleType)

	Df	Sum of Sqs	R^2	F	Pr(>F)
Model	1	4175	0.041	0.9931	0.049
Residuals	23	96687	0.959		
Total	24	100861	1.00		

adonis2(formula = sub_dist ~ Fe_supplementation, data = sub_meta.GVC)

	Df	Sum of Sqs	R^2	F	*Adjusted-p*
Model	1	6202	0.104	1.6203	0.024
Residuals	14	53591	0.896		
Total	15	59793	1.00		

adonis2(formula = sub_dist ~ Fe_supplementation, data = sub_meta.surface)

	Df	Sum of Sqs	R^2	F	*Adjusted-p*
Model	1	2317	0.142	1.163	0.024
Residuals	7	13943	0.857		
Total	8	16261	1.00		

Biology Open • Supplementary information

Table S7. Type II ANOVA results for the effects of coral species, sample type, and iron supplementation on microbial alpha diversity (ExpShannon). The linear model was fitted on log-transformed exponential Shannon diversity values, with factors including the interaction between species and sample type (interaction), Fe supplementation (Fe_supplementation), and their interaction.

TYPE II ANOVA

aov(log(ExpShannon) ~ interaction * Fe_supplementation, data = div)

	**Df**	**Sum of Sqs**	**F**	**Pr(>F)**
Interaction	3	20.153	28.705	< 0.001
Fe_supplementation	1	1.892	8.083	< 0.01
Interaction:Fe_supplementation	3	3.320	4.729	< 0.001
Residuals	44	10.297		

Biology Open • Supplementary information
